# Carbon nanotube nanocomposite scaffolds: advances in fabrication and applications for tissue regeneration and cancer therapy

**DOI:** 10.3389/fbioe.2023.1299166

**Published:** 2023-12-21

**Authors:** Andy Shar, Angela Shar, Daeha Joung

**Affiliations:** ^1^ Department of Physics, Virginia Commonwealth University, Richmond, VA, United States; ^2^ College of Medicine, University of Florida, Gainesville, FL, United States; ^3^ Massey Cancer Center, Virginia Commonwealth University, Richmond, VA, United States

**Keywords:** carbon nanotube, tissue engineering, regenerative medicine, cancer therapy, 3D printing, conductive scaffold

## Abstract

Carbon nanotube (CNT) nanocomposite scaffolds have emerged as highly promising frameworks for tissue engineering research. By leveraging their intrinsic electrical conductivity and valuable mechanical properties, CNTs are commonly dispersed into polymers to create robust, electrically conductive scaffolds that facilitate tissue regeneration and remodeling. This article explores the latest progress and challenges related to CNT dispersion, functionalization, and scaffold printing techniques, including electrospinning and 3D printing. Notably, these CNT scaffolds have demonstrated remarkable positive effects across various cell culture systems, stimulating neuronal growth, promoting cardiomyocyte maturation, and facilitating osteocyte differentiation. These encouraging results have sparked significant interest within the regenerative medicine field, including neural, cardiac, muscle, and bone regenerations. However, addressing the concern of CNT cytotoxicity in these scaffolds remains critical. Consequently, substantial efforts are focused on exploring strategies to minimize cytotoxicity associated with CNT-based scaffolds. Moreover, researchers have also explored the intriguing possibility of utilizing the natural cytotoxic properties of CNTs to selectively target cancer cells, opening up promising avenues for cancer therapy. More research should be conducted on cutting-edge applications of CNT-based scaffolds through phototherapy and electrothermal ablation. Unlike drug delivery systems, these novel methodologies can combine 3D additive manufacturing with the innate physical properties of CNT in response to electromagnetic stimuli to efficiently target localized tumors. Taken together, the unique properties of CNT-based nanocomposite scaffolds position them as promising candidates for revolutionary breakthroughs in both regenerative medicine and cancer treatment. Continued research and innovation in this area hold significant promise for improving healthcare outcomes.

## 1 Introduction

Tissue regeneration and cancer therapeutics are two critical areas in modern medicine that require innovative approaches to address various challenges. Over the past decade, carbon nanotube (CNT) nanocomposite scaffolds have emerged as promising remedies for addressing these challenges. With remarkable attributes, including exceptional mechanical strength, electrical conductivity, and biocompatibility, these scaffolds have captured considerable interest for their potential roles in advancing both tissue regeneration and cancer therapy. Their distinctive qualities position them as prime contenders across a spectrum of applications within the realm of regenerative medicine.

**FIGURE 1:**
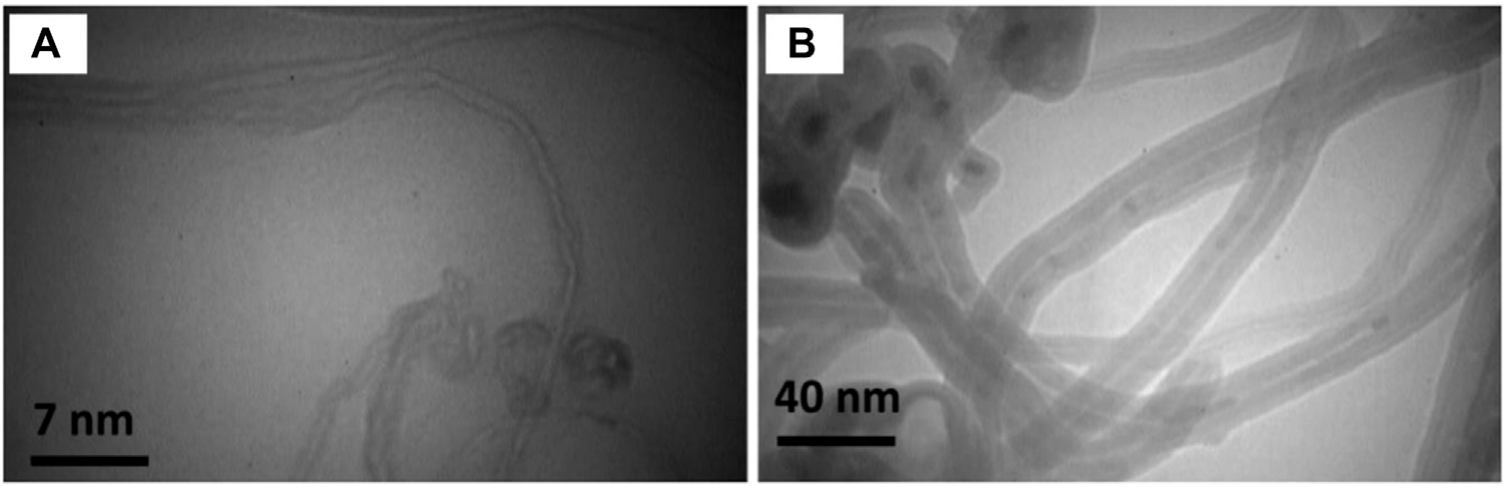
Transmission electron microscopy (TEM) characterization of **(A)** SWCNTs and **(B)** MWCNTs. Differences in tube diameters, aspect ratios, and layering arrangements between SWCNT and MWCNT contribute to varying biochemical properties and potential applications in bio-nanotechnology. Reprinted with permission from Zeinabad et al. (2016). Copyright (2016) Springer Nature.

Structurally, CNTs are cylindrical nanostructures composed of sp^2^ hybridized carbon atoms arranged in a hexagonal lattice, forming seamless tubes with nanometer-scale diameters (Dresselhaus et al., 2000; Gupta et al., 2019; Soni et al., 2020). Single-walled carbon nanotubes (SWCNTs) may be treated as a two-dimensional graphene sheet rolled into a hollow cylinder. Multi-walled carbon nanotubes (MWCNTs) consist of a series of concentric single-walled nanotubes positioned in a tube-in-tube structure. Nanotubes are either chiral or achiral; achiral nanotubes can be further divided into the zigzag configuration and the armchair configuration depending on the specific arrangement of carbon atoms. Moreover, SWCNTs may be either semiconducting or metallic depending on small differences in chiral angle or diameter. SWCNTs generally have smaller diameters than MWCNTs. This difference in diameter affects their surface area-to-volume ratio and influences interactions with cells and biomolecules (Figure 1) (Zeinabad et al., 2016).

Both SWCNTs and MWCNTs possess exceptional mechanical, thermal, and electrical properties, making them highly attractive for various applications, including tissue regeneration scaffolds. MWCNTs tend to exhibit better mechanical properties, such as higher tensile strength and stiffness, due to their multiple layers. This can make them more suitable for providing structural support in tissue engineering scaffolds. CNTs exhibit one of the highest tensile strengths (∼0.85 GPa) and Young’s moduli (∼34.65 GPa) among known materials, enabling them to withstand significant forces without deformation or fracture (Kim et al., 2017; Kumar et al., 2021). Thermal conductivities of MWCNTs and SWCNTs range from ∼2,000 to 3,000 W/mK, allowing for rapid heat transfer through CNT-based bioengineering systems (Lee et al., 2022). Additionally, CNTs exhibit outstanding electrical conductivity (∼10^6^–10^7^ S/m) due to the delocalized π-electron system along their tubular structure, allowing them to transport electrical charges efficiently (Wang and Weng, 2018; Gupta et al., 2019). This electrical conductivity can be harnessed for electrical stimulation or sensing applications within tissue engineering constructs. As a result, since electrical charges are key for cell-to-cell communication in neurite and cardiomyocyte systems, CNT scaffolds promote proliferation and stem cell differentiation within these tissue types (Burnstine-Townley et al., 2020; Nekounam et al., 2021). MWCNTs, with their larger diameter and multiple layers, may have a higher loading capacity for therapeutic molecules, growth factors, or drugs. This could be advantageous for controlled-release applications in tissue engineering. Therefore, it is important to note that both SWCNTs and MWCNTs have unique properties that can be harnessed for various applications in tissue engineering. The choice between these two types of nanotubes depends on the specific requirements of the tissue engineering application, the desired properties of the scaffold, and the potential benefits and challenges associated with each type.

An important aspect of CNT-based nanocomposites that sets them apart from other conductive nanofillers is the low % loadings needed for the percolation phenomenon. This refers to a process where the electrical conductivity of these composites increases dramatically around certain concentrations of carbon fillers, as opposed to a linear increase. This sudden boost in conductivity can make the composite shift from nearly insulating to highly conductive. The percolation threshold is the percentage of conductive filler at which they start forming a network, rather than being isolated within the insulating matrix. Electrical current is allowed to flow freely, and the rapid conductivity increase (by a factor ≥ 10^3^) signifies a shift in the composite’s overall properties from those of the insulating phase to those of the conducting phase. One of the factors affecting the percolation threshold is the geometrical structure of the composite, specifically the aspect ratio (length-to-diameter ratio) of the nanofiller. The long cylindrical microstructure of CNTs gives them a high aspect ratio (>100), allowing for low percolation thresholds of 0.1%–1% (Li et al., 2007).

The incorporation of CNT into tissue regeneration studies can be classified into two types: 1) as a conductive filler in polymeric scaffolds, and 2) as a nanomaterial drug engineered to directly infiltrate target cells (Eivazzadeh-Keihan et al., 2019). In the first application, CNT is dispersed into a polymer matrix to impart its favorable conductive and mechanical properties. These polymers include natural hydrogels [alginate (Alg), gelatin (Gel), collagen (Col), agarose (AG), chitosan, *etc.*,] as well as synthetic biocompatible polymers [polycaprolactone (PCL), polyethylene glycol (PEG), polylactic acid (PLA), poly-lactide-co-glycolide (PLGA), polydimethylsiloxane (PDMS), *etc.*,] (Amiryaghoubi et al., 2021). MWCNTs, with their larger diameter and mechanical strength, could find use in structural scaffolds for tissue engineering, where mechanical support is crucial. In the second application, CNT is loaded with drug molecules, often with anti-inflammatory or growth factor-like effects (Saleemi et al., 2020). Due to the hydrophobicity of CNT, the drug carrier system easily passes through the cell membrane. Often, SWCNTs are preferred for this application requiring precise control over nanoscale interactions, such as targeted drug delivery, due to their larger specific surface area, which may be attributed to the smaller diameter of SWCNTs (∼0.8 nm) compared to MWCNTs (∼11 nm) (Öner et al., 2018; Karimzadeh et al., 2021).

**SCHEME 1:**
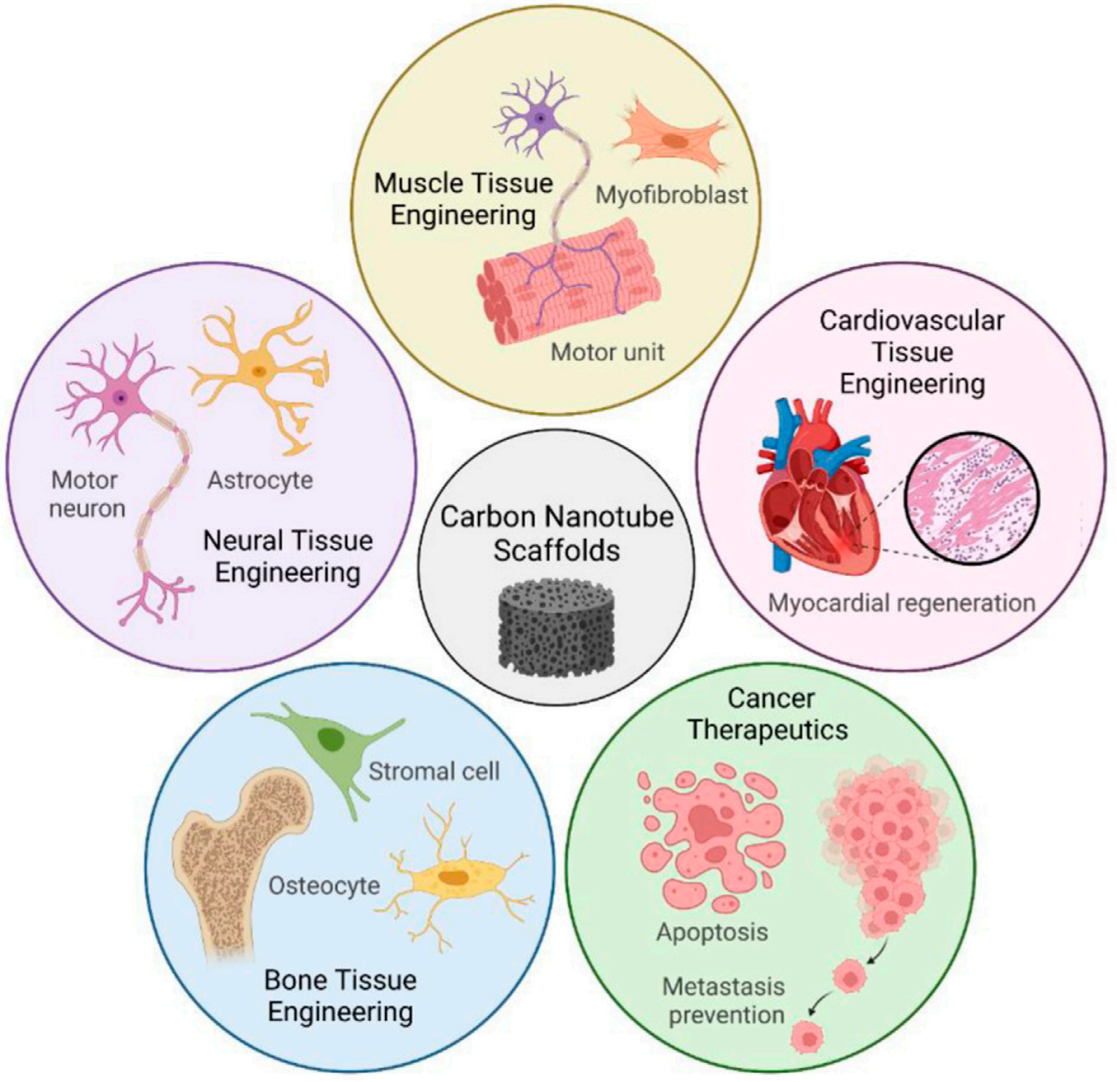
CNT-based scaffolds have a diverse range of applications in tissue engineering. Conductive and biocompatible CNT scaffolds promote cell attachment, differentiation, and proliferation in nervous, cardiovascular, muscle, and bone tissue. Recent discoveries also harness the cytotoxicity of CNTs as the foundation for next-generation cancer therapeutics. Parts of Scheme 1 were created with Biorender.com.

This review will focus on the former application, of CNT-based scaffolds, because 3D-microengineered scaffolds are uniquely able to induce tissue regeneration in three dimensions (Scheme 1). Moreover, we will explore how CNT itself imparts favorable mechanical and biological properties to CNT nanocomposites, and how these properties shape the response of cells and promote regeneration. Nevertheless, the latter application of CNT in drug carrier systems is still relevant, and more information on this topic can be found in other studies (Yan et al., 2019; Mangla et al., 2020; Saleemi et al., 2020). Neural, cardiac, muscle, and bone tissue engineering are four areas of research where CNT-based scaffolds have emerged as promising tools. In relation to neurites, cardiomyocytes, and myocytes, electrical conductivity in a scaffold promotes action potentials and intracellular communication (Dong et al., 2020; Kunisaki et al., 2021; Scott et al., 2021). Concerning osteocytes, CNT promotes protein adsorption and improves scaffold rigidity to match the native bone environment better (Patel et al., 2020).

Despite these positive attributes, the cytotoxicity of CNTs is a critical aspect that must be carefully considered in the context of tissue regeneration and cancer therapeutics. Studies have indicated that the cytotoxicity of CNTs can vary depending on factors such as their length, diameter, surface functionalization, and concentration. Unmodified CNTs, especially those with longer lengths and higher aspect ratios, have been shown to induce oxidative stress, inflammation, and damage to cellular membranes, leading to compromised cell viability and function (Huang, 2020). This paper will delve into strategies to reduce cytotoxicity, such as surface functionalization of CNTs. Interestingly, the cytotoxicity of CNTs can also be leveraged for potential applications in cancer therapeutics. This review will explore this property of CNT, as well as less well-known properties, such as high photothermal conversion efficiency and Joule heating effects. Specifically, these properties set CNT-based scaffolds apart from other nanofiller-based scaffolds as attractive candidates for localized heating and targeted cancer therapy (Faraji Dizaji et al., 2020).

## 2 Fabrication of CNT-based scaffolds

### 2.1 CNT dispersion in nanocomposites

Due to strong Van der Waals and π-π interactions between carbon nanotubes, they tend to aggregate easily and are hard to disperse within a polymer matrix (Ma et al., 2010). MWCNTs, due to their multiple layers, might have comparatively better dispersion properties than SWCNTs. There are three main strategies to disperse CNT: solution mixing, melt mixing, and shear mixing. These strategies have been chosen to explore because they are widely used and compatible with a variety of polymers, from epoxy resins to hydrogels to silicone-based polymers (Singh et al., 2019; Patil et al., 2021; Shar et al., 2023).

By far, the most common strategy employed to disperse CNT in polymer matrices such as, but not limited to PCL, PEG, PLA, PDMS, Gel, Alg, AG, and cellulose, is solution mixing (Velasco-Mallorquí et al., 2020; Jiang et al., 2021; Shar et al., 2023; Vasconcelos et al., 2023). In solution mixing, CNT and polymer are dispersed separately/together into a common organic or aqueous solvent, including water, acetone, isopropanol, butyl acetate, trifluoroethanol, *etc.* The solvent is subsequently evaporated through heat or vacuum to achieve a well-dispersed solid CNT nanocomposite. Solution mixing is mainly achieved through ultrasonication, the use of ultrasound energy to agitate particles in a solution. For example, CNT was dispersed into a series of polyurethane solutions in aqueous acetic acid through ultrasonication (Ahmadi et al., 2021). A common strategy in CNT ultrasonication is to first sonicate a stand-alone solution of CNT to achieve a well-dispersed solution (Mombini et al., 2019). Then, the sonicated solution is added to a solution of polymer, and the mixture is sonicated again to achieve a homogenous dispersion of CNT and polymer in the chosen solvent. Advantages of ultrasonication and solution mixing include low cost and ease of use. However, excessive or prolonged sonication can damage CNTs, causing defects and converting them into amorphous carbon nanofibers, negatively affecting the electrical and mechanical properties of CNT/polymer composites (Arrigo et al., 2018).

Shear mixing is a broad term referring to techniques in which a mixing blade or impeller creates intense turbulence to disperse immiscible chemicals (Kundalwal and Rathi, 2020). Ball milling is a high-energy technique in which collisions between small rigid spheres generate extreme pressures capable of dispersing CNT within matrix materials. In one instance, ball milling was used in conjunction with ultrasonication to toughen a bioactive glass nanocomposite scaffold with CNT (Liu et al., 2015). An ultrasonicated CNT-ethanol solution was mixed with bioactive glass nanoparticles, and the wet mixture was subject to a high energy ball milling machine. Following evaporation of ethanol, the powder was selectively sintered through a computer-controlled mechanism to fabricate the three-dimensional (3D) scaffold. While ball milling is a process easily adapted for large-scale production, it suffers from issues of insufficient dispersion and possible contamination from milling media (Lyu et al., 2017). Calendering is another type of shear mixing in which a mixture of CNT and polymer is forced between rotating rollers. A high shear stress is achieved through this method, which helps to disentangle CNT bundles. However, there are several limitations to calendering, such as its cost (∼$5000–$50,000 per machine). The minimum gap between rollers is ∼ 1–5 μm, which is larger than the diameter of individual CNTs (Ma et al., 2010). Thus, while calendering may break down large agglomerates of CNTs at a sub-micron level, individual CNTs may be left disentangled. Air bubbles are also easily entrapped within the rolling system, potentially creating homogeneity issues in the final CNT nanocomposite mixture (Atif and Inam, 2016).

One subtype of shear mixing is melt mixing; for the production of CNT-thermoplastic polymer composites, melt mixing is a practical and economical method (Banerjee and Dutta, 2019; Sui et al., 2019). Examples of thermoplastics for which melt mixing is a viable option include polymethyl methacrylate (PMMA), acrylonitrile butadiene styrene (ABS), nylon, polycarbonate (PC), and PLA. The technique is particularly suitable for polymers that are insoluble in common organic solvents (Abzan et al., 2021). A Banbury mixer or an extruder machine is used to combine a polymer matrix with CNTs before exposing the mixture to shear pressures at high temperatures (Rosemary, 2022). As a result, CNT bundles inside the polymer matrix are broken up and dispersed. Choosing less viscous thermoplastics, such as those with shorter polymer chains, allows for a more accessible combination with nanotube bundles as the temperature rises. The blending process requires no solvents, supports a bulk volume of polymers, is affordable, and has few negative environmental consequences. Melt mixing yields CNT nanocomposites with diverse applications in tissue engineering. For example, SWCNT-PLA filaments were synthesized using the melt mixing technique and showed no cytotoxicity even at very high SWCNT loadings (40%) (Kim et al., 2019). This illustrates extensive encapsulation of CNT moieties by the PLA polymer matrix, resulting in an overall nontoxic material. In other studies, CNT-PMMA composites have been used to improve cytocompatibility and osseointegration (Wang et al., 2019). At a loading of only 1% MWCNT, the bone ingrowth ratio increased by 42.2% at 12 weeks post-surgery. However, there are drawbacks to the process, as the use of high shear force and high temperature can potentially damage the CNTs and polymer chains (Mohd Nurazzi et al., 2021).

*In-situ* polymerization is a versatile technique employed in the fabrication of CNT-polymer composites for tissue engineering applications. *In-situ* polymerization typically involves the introduction of monomers and CNTs into a reaction medium, followed by the initiation of polymerization (Chazot et al., 2020; Maharjan et al., 2020). This can be achieved through various methods, including thermal, chemical, or radiation initiation (Chan et al., 2019). The CNTs, well-dispersed in the reaction mixture, become embedded in the growing polymer chains, leading to a seamless integration of nanotubes within the polymer matrix. One study prepared CNT-hydrogel composites from *in situ* polymerization of PEG around a CNT meshwork (Ye et al., 2021). Culturing adult neural stem cells on the composites results in an elevated ratio of neurons to astrocytes (∼2× increase over control glass substrates). Furthermore, primary hippocampal neurons grown on these composites retain both morphological synaptic features and exhibit neuronal network activity, as assessed through spontaneous calcium oscillations. Another study developed mechanically robust and biocompatible scaffolds using sunflower oil-modified hyperbranched polyurethane (HBPU) combined with functionalized multi-walled carbon nanotubes (f-MWCNTs). The scaffolds demonstrated sufficient strength (39 ± 1.65 MPa) after 60 days of *in vitro* biodegradation. Additionally, the interconnected porous nanocomposite with a pore size of 200–330 μm showed superior proliferation and adherence of osteoblast cells, affirming its potential as a non-toxic biomimetic scaffold for bone tissue engineering. One of the key advantages of *in-situ* polymerization is the uniform dispersion of CNTs throughout the polymer matrix. Monomer-based mixtures are usually less viscous than polymer-mixtures, improving separation of CNT. Moreover, *in-situ* polymerization allows for strong interfacial interactions between the CNTs and the polymer matrix. The simultaneous formation of polymer chains around the nanotubes improves adhesion, reducing the likelihood of phase separation and enhancing the overall stability of the composite. However, the disadvantages of *in-situ* polymerization include a short working period (5–30 min), constraints on useable polymers, and highly complex steps (Chen et al., 2020; Zamiri and Haseeb, 2020; Pereira et al., 2022).

CNT dispersion remains at the forefront of engineering CNT-polymer systems with optimal homogeneity. While organic solvents are still the most popular choice for CNT-polymer solution mixing, researchers look towards green, alternative solvents that are more environmentally friendly to produce and release less harmful vapors, such as 1,3-dioxolane and ionic liquids (Moscoso et al., 2014; Nguyen and Shim, 2015). Moreover, more research should be directed towards molecular dynamic (MD) simulations, which have emerged as a powerful tool to study CNT dispersions on a molecular level. By being able to screen potential surfactants and solvent mixtures, researchers can be more efficient in finding the proper combination of surfactant and solvent to disperse CNT into polymer networks. Finally, while solution mixing remains popular, melt-mixing and shear mixing are viable options for thermoplastic polymers and polymers resistant to dissolution. Progress in the dispersion of CNT-polymer systems for tissue engineering is shown in Table 1.

**TABLE 1:** Progress in the dispersion of CNT-polymer systems for tissue engineering.

Approach	Description	Applicable polymers	Advantages	Disadvantages
Solution mixing	CNT and polymer dispersed in an organic or aqueous solvent, often through ultrasonication; followed by solvent evaporation	PCL, PEG, PLA, PDMS, Gel, Alg,Col, chitosan, hydroxyapatite	- Low cost and ease of use	- Excessive or prolonged sonication can lead to CNT defects and amorphous carbon nanofiber formation
			- Compatible with a variety of polymers	- Extended working time needed to evaporate solvent homogenously
			- Effective for a wide range of solvents (water, acetone, isopropanol, *etc.*)	
Shear mixing	Any technique involving mixing blades or impeller to create turbulence. Examples include ball milling, calendaring, and shear mixing	PDMS, PMMA, ABS, PCL, nylon	- Ball milling is adaptable for large-scale production	- Potential for insufficient dispersion and contamination from milling media
			- Calendering can disentangle CNT bundles with high shear stress	- Cost may be prohibitive
			- Practical and economical for thermoplastic polymer composites	- Potential for entrapped air bubbles, reducing homogeneity in the final CNT nanocomposite
			- No solvents required, supporting a bulk volume of polymers	- High shear force and temperature can potentially damage CNTs and polymer chains
			- Few environmental consequences	
*In-situ* polymerization	Polymer chains are formed directly within the presence of carbon nanotubes	PEG, PU, PMMA, PS, HDPE	- Uniform dispersion of CNTs throughout the polymer matrix	- Short working period (5–30 min)
			- Strong interfacial interactions between the CNTs and the polymer matrix	- Constraints on useable polymers
				- Highly complex steps
Molecular dynamics (used in conjunction with other methods)	Simulations to study CNT dispersions on a molecular level; screening of potential surfactants and solvent mixtures	Various	- Powerful screening tool to narrow choices for solvent	- Theoretical approach may not fully represent real-world conditions
			- May be used to engineer and predict complex solvent blends	

### 2.2 CNT functionalization

To enhance dispersion within nanocomposite scaffolds, CNTs have undergone functionalization. [CNT may also be used to immobilize/encapsulate peptides, genetic material, and pharmaceutical drugs, but these applications are outside of this article’s focus on CNT-based tissue engineering scaffolds (Klumpp et al., 2006; Nagaraju et al., 2015; Dubey et al., 2021).] Polar functional groups are often covalently attached to CNT to overcome their primarily hydrophobic nature and improve dispersion in water or hydrogel (Dubey et al., 2021). In the fabrication of CNT-PCL electrospun scaffolds for bone tissue engineering, amine-functionalized SWCNT was sonicated in dimethylformamide (DMF) to obtain a stable dispersion in concentrations of 0–0.5 wt%; this SWCNT-DMF dispersion was then added to a separate 10% w/v PCL-dichloromethane (DCM) solution (Tohidlou et al., 2019). The amino groups increased the polarity of the SWCNT and enabled successful interface with the ester groups of PCL. In another study, SWCNT was functionalized with carboxylic groups (COOH) (Bahrami Miyanji et al., 2022). The enhanced hydrophilicity of the SWCNT enabled direct addition to a chitosan-gelatin system dissolved in a polar DCM/trifluoroacetic acid (30:70 v/v) mixture. The carboxylic acid groups strongly engaged in dipole-dipole interactions with the trifluoroacetic acid solvent, improving CNT dispersion in 0–1 wt% concentrations. The resulting chitosan-gelatin-CNT scaffolds displayed increased porosities suitable for cartilage tissue engineering applications.

Recent advances have pointed to noncovalent functionalization as a possible tool to improve the water dispersibility of CNT and allow for the fabrication of more hydrophilic scaffolds. A PEG hydrophilic linker with a pyrene moiety bonded firmly to the delocalized surface of CNT, while significantly improving its dispersion in water (Assali et al., 2022). The study also measured the polydispersity index, which measures the uniformity of molecular mass in a polymer sample, with 0.0 representing a completely homogenous sample, and 1.0 representing a highly diverse sample with distinct particle sizes. After functionalization with pyrene, the polydispersity index of both SWCNT and MWCNT decreased, indicating less agglomeration (0.599 → 0.283, 0.462 → 0.263, respectively). Engineering connective tissues generated with the functionalized CNTs demonstrated increases in electrical conductivity with a reduction in tissue fibrosis (from 2 to 15 S/m) (Assali et al., 2022). Interestingly, concentrations of 0.1% and 0.05% of CNT led to disorganization of the extracellular matrix (ECM) and the onset of fibrosis, while lower concentrations, such as 0.025%, displayed low toxicity. Thus, it appears that colocalization of CNT with cells can be disadvantageous at higher concentrations. Ensuring cytotoxicity is controlled when working with CNT-based scaffolds remains a relevant issue.

Functionalization of CNT with polymers to produce polymer-grafted CNTs can aid in stronger chemical interactions with a polymer matrix (Díez-Pascual, 2021). Example reactions include coupling and nucleophilic addition reactions, cycloaddition reactions, and amide/ester linkages. Of these, organometallic approaches have been reported as effective avenues for CNT functionalization. One notable application of this epoxy functionalization method involves the grafting of an aminated polyphenylene sulphide (PPS-NH2) derivative onto epoxy-functionalized SWCNTs (Díez-Pascual and Naffakh, 2012). n-butyllithium is first added to a SWCNT suspension in toluene, creating a reactive mixture that yields a degree of grafting of 25%. In a parallel study, the reaction between acyl-chloride-modified MWCNTs and polystyryllithium anions achieved a notably high grafting level, close to 40% (Wu et al., 2003). These organometallic functionalization processes exemplify a versatile method for modifying CNTs, offering opportunities for further derivatization and enhancing the compatibility of CNTs with various polymers.

An alternative strategy to fabricate polymer-grafted carbon nanotubes involves the use of reversible addition–fragmentation chain transfer (RAFT) polymerization (Hosseinzadeh et al., 2020; Eskandari et al., 2021). This approach offers precise control over the molecular mass of the polymer resulting in a narrow molecular mass distribution. RAFT polymerization can be implemented under mild conditions for various techniques such as emulsion, bulk, or suspension polymerization. Notably, RAFT polymerization is versatile, accommodating a wide range of water-soluble monomers. In one instance, RAFT polymerization was used to graft poly (N-isopropylacrylamide) (PNiPAAM) onto MWCNTs (Hong et al., 2005). TEM images illustrated that the MWCNT-g-PNiPAAM nanocomposite exhibited excellent solubility in water, chloroform, and tetrahydrofuran, along with effective disentanglement of tube bundles. This process has been repeated to form PS-grafted carbon nanotubes that are soluble in tetrahydrofuran, dichloromethane, and chloroform (Li et al., 2005). In another instance, 2,2-azobisisobutyronitrile (AIBN) was employed as an initiator for grafting polystyrene (PS) chains onto MWCNTs via RAFT polymerization (Cui et al., 2004). TEM micrographs of the samples verified the development of a core–shell nanostructure. The utilization of RAFT polymerization in these studies showcases its efficacy in achieving controlled and well-defined grafting of polymers onto CNTs, opening avenues for the tailored design of advanced nanocomposites with tunable properties.

Functionalization of CNTs is an active field of research with potential to improve CNT biocompatibility, dispersibility, and chemical properties. More research should be directed towards functionalizing CNT for biocompatible dispersion in water. As hydrogels such as sodium chitosan, Alg, Gel, and more are common as tissue engineering construct materials, new ways to disperse CNT in water would improve the quality of the final CNT-hydrogel material. As highlighted previously, aromatic surfactants such as pyrene have shown promise for these aqueous dispersions, but more environmentally friendly surfactants should also be explored.

### 2.3 Strategies of scaffold fabrication

Many avenues exist to fabricate CNT-based tissue engineering scaffolds, including 1) inkjet printing, 2) electrospinning, 3) direct link writing (DIW) or extrusion-based 3D printing, 4) stereolithography and 5) freeze-drying. These five techniques are the most widely used techniques and have been applied to CNT-elastomers, CNT-polyesters, CNT-hydrogels, CNT-mineral composites, and more (Shokri et al., 2015; Ahadian et al., 2017; Jiang et al., 2021).

**FIGURE 2:**
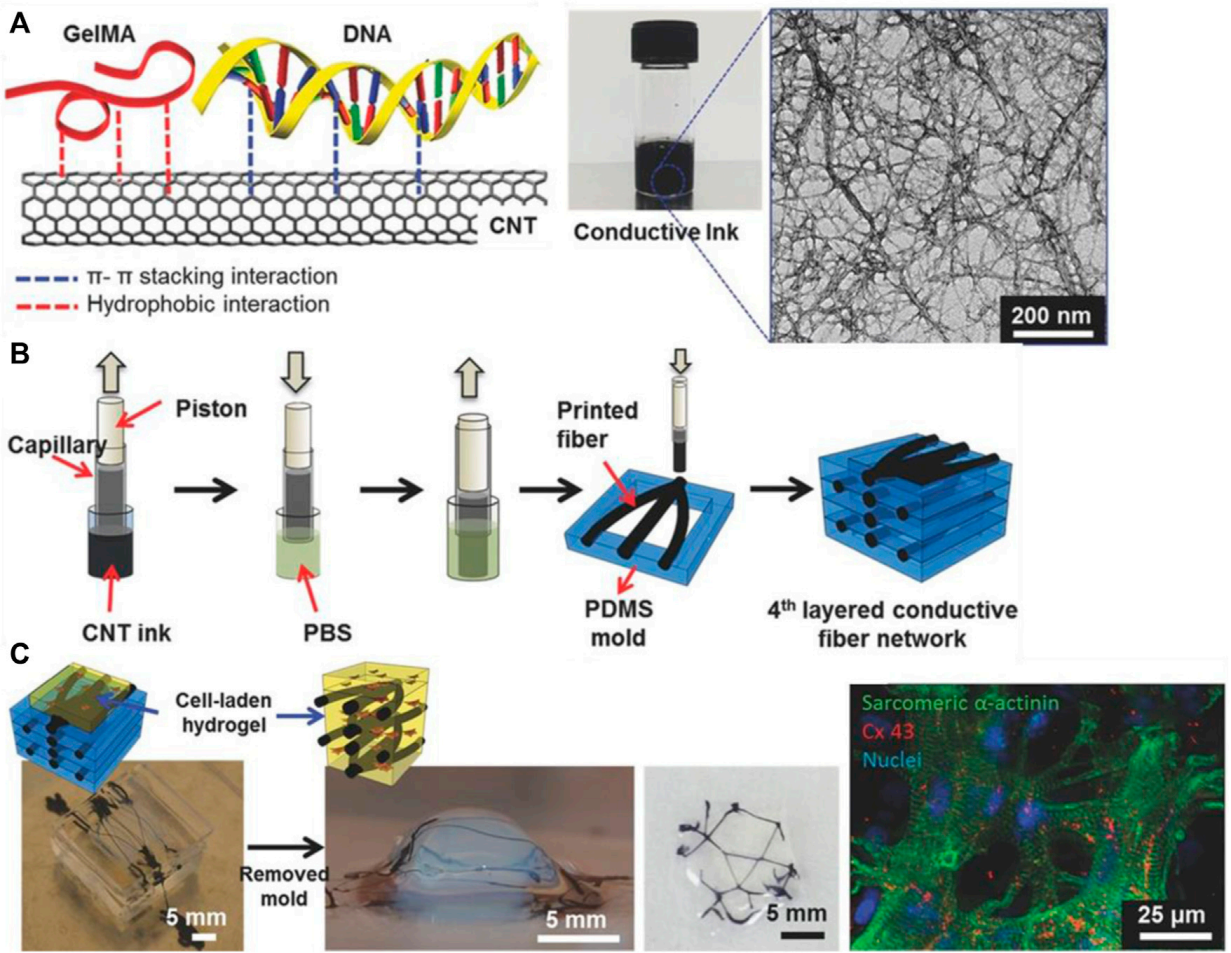
**(A)** Chemical makeup of DNA-assisted CNT-GelMa conductive ink. Aromatic interactions between bases of DNA and the surface of CNTs facilitate dispersion. **(B)** 3D-printing apparatus to construct a conductive fiber network of CNT-GelMa. **(C)** Imaging of cell survival and proliferation along conductive CNT network. Reprinted with permission from Shin et al. (2016). Copyright (2016) Wiley-VCH GmbH.

Inkjet printing is a simple 2D technique in which droplets of CNT-based ink are expelled onto a substrate. While the technology itself is not novel, new compositions of CNT-based inks have been discovered to improve the viability of inkjet printing in tissue engineering. A unique approach used DNA as a biosurfactant to disperse MWCNT within gelatin methacryloyl (GelMa), a gelatin-derived bioactive matrix, and HA, a natural glycosaminoglycan and lubricant (Shin et al., 2016) (Figure 2). GelMa/DNA-wrapped MWCNT ink was synthesized and printed on culture glass, cellulose, PEG, and other diverse substrates. Free-standing, flexible, and resilient films with thicknesses of ∼10 μm were also fabricated. To evaluate cytocompatibility, cardiac fibroblasts were cultured onto printed CNT ink patterns, demonstrating high viability, metabolic activity, and normal phenotypes. While inkjet printing is powerful, it also has precise requirements, such as (i) stability of nanoparticle dispersion in solution and (ii) proper viscosity of ink. For the former requirement, good homogeneity and dispersion of CNT into the ink are key, and methods such as ultrasonication and the use of surfactant are necessary. For the latter requirement, parameters such as solvent vapor pressure, percentage loading of CNT, and diameter of the nozzle tip must be controlled. As inkjet printing is a high-resolution technique (∼10–50 μm), CNT agglomeration can clog nozzle tips and be detrimental to consistent results. Nevertheless, inkjet printing remains a useful technique for CNT-bioink printing because it is low-cost and requires no prefabrication of masks or templates (Kholghi Eshkalak et al., 2017).

In the electrospinning process, a spinning syringe tip is used to release a charged jet of polymer solution or melt. The polymer solution hardens by solvent evaporation to produce fibers with a consistent diameter and porosity. An advantage of electrospinning is its ease of use, as it only requires an electrospinning apparatus and a CNT-dispersed ink with a volatile solvent such as chloroform, trifluoroethanol, DMF, *etc.* A polyurethane/chitosan/CNT nanofibrous scaffold was electrospun and confirmed as viable substrates for cardiac myocyte tissue (Ahmadi et al., 2021). In another study, electrospun PCL/Gel/CNT yarns were used to fabricate fabric-like scaffolds with good biocompatibility and the ability to guide cell elongation (Jiang et al., 2021). The resulting fibers possessed similar mechanical properties to native blood vessels and hold potential for applications in vascular tissue regeneration. Researchers have also investigated adding laminin, a neurite promoting protein, to modify PLGA/CNT scaffolds. To this end, a simultaneous PLGA electrospinning and MWCNT electrospraying procedure was followed. A solution of MWCNT in ethanol and PLGA solution in hexafluoroisopropanol (HFIP) were loaded into two syringes with separate syringe pumps.

Following the electrospinning, the PLGA/CNT scaffolds were incubated with a laminin solution and easily obtained a laminin coating. The prior electrospinning process ensured the uniform structural integrity of PLGA/CNT without disturbing the laminin coating. A recent study investigated a solvent-free aqueous method to fabricate conductive CNT/silk composite scaffolds by electrospinning (Zhao et al., 2020). Additionally, the aligned morphology of the CNT/silk scaffolds promoted the creation of gap junctions by mimicking the natural environment of the myocardium. Notably, the alignment of CNT fibers confers anisotropic conductivity, which imitates the striated, parallel morphology of cardiac muscle.

Despite its simplicity, electrospinning has some drawbacks, such as the inability to create conduits with multiple biomaterials, including cells, neurotrophic factors, or any other bio-cues inside the polymer matrix. The high operation voltages of ∼10 kV and agitation of the scaffold inks prevent the addition of mechanically sensitive materials, including but not limited to living cells. Moreover, the streamlined process is not easily customized, resulting in difficulty in printing structures with complex topologies (Nadaf et al., 2022).

To overcome these limitations, extrusion-based 3D printing (including bioprinting) has emerged as an additive manufacturing technique to construct micro-engineering CNT-based scaffolds. In extrusion-based 3D printing, a viscous CNT-polymer ink is placed into a syringe. Air pressure from a compressor pushes the ink through a narrow tip, extruding directly onto the substrate. For extrusion printing to be successful, the ink must be thixotropic, flowing when subject to high shear stress, but returning to a more viscous state when the force is removed. This enables smooth outflow from the nozzle tip while maintaining its shape once the ink is deposited onto the substrate. In one instance, CNT-PCL ink with chloroform as the solvent was extruded through a tip with an inner diameter of ∼560 μm to form grid-like scaffolds (Ho et al., 2017). A slight increase in the proliferation of H9c2 cells (embryonic rat cardiomyocyte line) was observed on the CNT-PCL scaffolds compared to pure PCL scaffolds (optical density increases from 0.225 → 0.275). However, enzymatic biodegradation studies conducted on these scaffolds showed a slight decrease in degradation rate in CNT-PCL samples. At 6 days, CNT-PCL scaffolds exhibited weight losses of only 60% compared to 80% for their PCL counterparts. This effect is useful as researchers can vary CNT percentage to adjust for specific tissue engineering applications. For example, a large injury to the myocardium may require a slower-degrading scaffold (higher CNT%) to allow more time for cell proliferation, while a smaller injury may require a faster-degrading scaffold (lower CNT%) so that the scaffold may break down simultaneously with the wound healing.

The advantage of extrusion-based 3D printing lies in the ability to create CNT-based scaffolds from scratch that precisely fit the patient’s needs. A conductive nanocellulose-CNT ink was 3D-printed to form neural guidelines with specially designed features such as nanotopography and electrical conductivity (∼3.8 × 10^−1^ S/cm). The guidelines were printed with a height of ∼10 μm, diameter of ∼300 μm, and showed uniform width and similar roughness. SH-SHY5Y human neuroblastoma cells were cultured onto the printed neural guidelines and proliferated further on the printed CNT guidelines compared to pure cellulose scaffolds. On a scaffold in which both cellulose and CNT guidelines were available, the cells committed to the CNT guidelines and ignored the pure cellulose material (Kuzmenko et al., 2018).

However, as with inkjet printing, extrusion-based 3D printing is limited by the need for control over the CNT-ink viscosity and degree of dispersion. The CNT-bioink must be thixotropic, or stable at rest and fluid when pressure is applied. This property enables the final scaffold to maintain form, while allowing for easy extrusion through the nozzle tip during printing. In the aforementioned study, rheology tests showed a negative linear trend in viscosity vs shear rate, indicating the CNT-nanocellulose ink was thixotropic (Kuzmenko et al., 2018). Moreover, the intense shear forces associated with extrusion-based 3D printing are detrimental to cell viability and limit the inclusion of living cells in the ink (Placone et al., 2020). New studies show that it is possible to 3D-print bio-inks, but more research is needed to incorporate both CNT and cells into the same ink for efficient 3D bioprinting of conductive ink (Li et al., 2020b).

**FIGURE 3:**
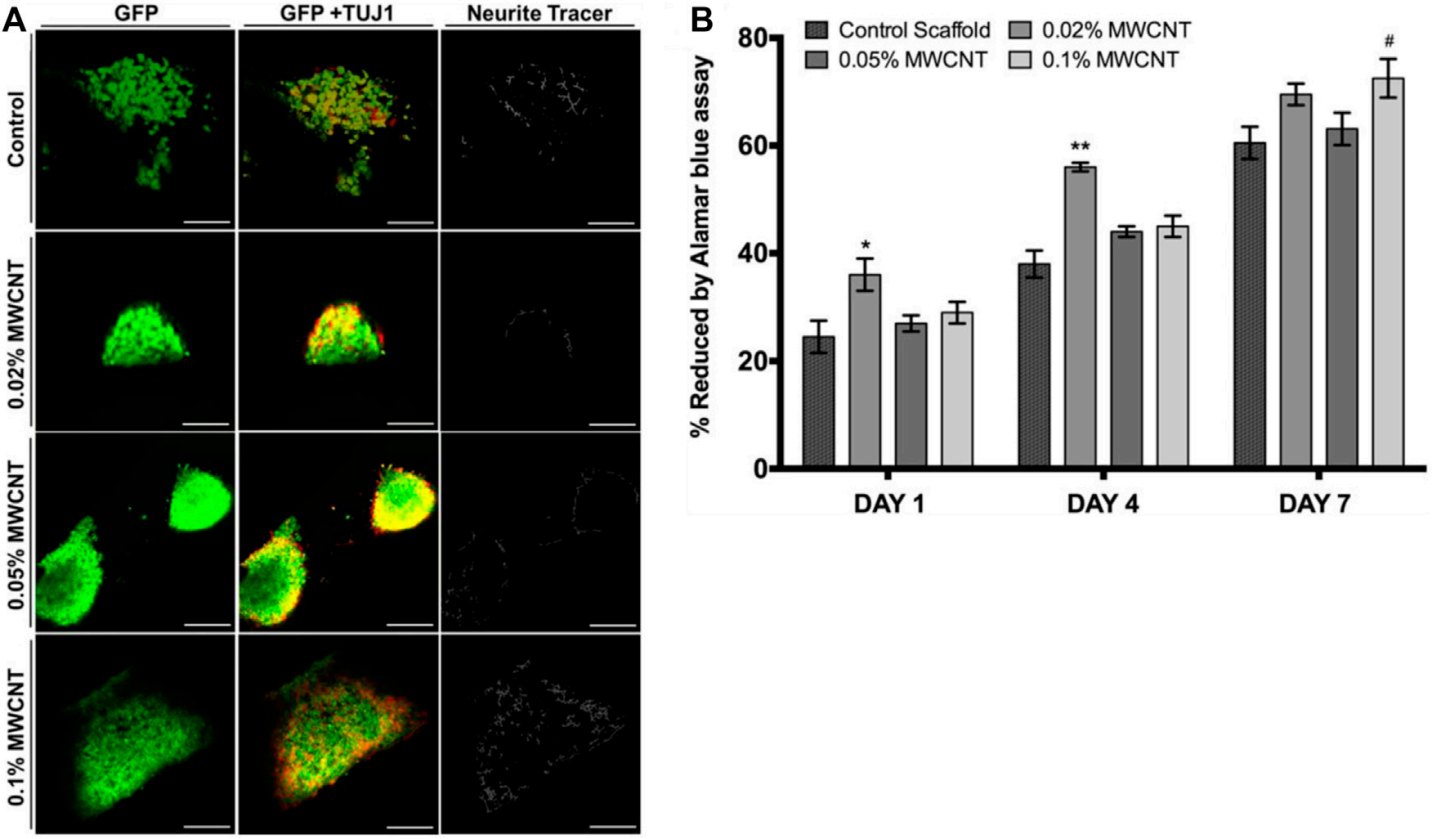
**(A)** Confocal micrographs of neural stem cells on MWCNT-PEGDA and pure PEGDA scaffolds after 8 days. Scale bar = 100 μm. In 0.1% MWCNT-incorporated scaffolds, mature neurite outgrowth increased, as quantified by NeuriteTracer. **(B)** Neural stem cell adhesion was evaluated by Alamar blue assay and showed positive results for all tested MWCNT loadings. Reprinted with permission from Lee et al. (2018). Copyright (2018) Institute of Physics.

Stereolithography (SLA) utilizes a photochemical process to cross-link polymers in a layer-by-layer nature. Through stereolithography, a 3D scaffold mold can be generated in computer-aided design (CAD) and parsed into 2D slice data files through computer software with predetermined layer thickness and resolution. An ultraviolet (UV) laser is then used to execute the files, crosslinking the polymer resin layer-by-layer until the desired topology is produced. Following printing, scaffolds are rinsed to remove uncured residue and cured with UV light to impart greater mechanical properties. As any remaining contaminants are detrimental to the biocompatibility of tissue engineering scaffolds, any scaffolds produced with stereolithography are also extracted thoroughly with organic solvents through a Soxhlet extraction apparatus (Liu et al., 2020b). Despite its advantages, SLA is limited to a narrow range of polymers that can be cured from a liquid solution of monomers. Moreover, UV radiation creates a harsh environment, limiting the use of biologically active substances within the polymer scaffold (Huang et al., 2020). Nevertheless, CNT-PEDGA scaffolds are one example of successful nerve regeneration scaffolds fabricated through stereolithography (Lee et al., 2018). A CNT-PEGDA ink was printed with a stereolithography setup with a laser spot diameter of ∼190 μm. A square pore scaffold geometry with a charge capacity of 2.21 ± 0.12 mC/cm^2^ was constructed and electrically stimulated. Significant increases in neural stem cell proliferation in 3D-printed scaffolds were observed in as little as 0.02% MWCNT loadings (Figure 3). Thus, SLA is a viable process for 3D-printing polymers such as PEGDA and GelMa, which require UV-activated crosslinking to cure.

Freeze drying, also known as lyophilization, is a widely used method in the fabrication of tissue engineering scaffolds. This technique involves removing water or other solvents from a frozen sample, leaving behind a porous structure that can serve as a scaffold for tissue growth (pore size = 40–200 μm). An advantage of the freeze drying is the ability to produces a porous structure that mimicks the natural extracellular matrix (ECM) of tissues. The permeable scaffolds facilitate nutrient and oxygen diffusion and support cell migration, proliferation, and tissue ingrowth. The freezing step in freeze-drying allows for control over the size and distribution of pores within the scaffold. This control is important as different tissues have varying requirements for pore size to support cell activities. In one study, MWCNT-curcumin-Col scaffolds were fabricated through freeze drying (Farshidfar et al., 2022). The optimal composition, determined to be 1% MWCNTs and 10% curcumin, resulted in scaffolds with the highest mechanical and physical properties. The composite scaffolds exhibited a significant increase in average pore size, transitioning from 67 ± 11 μm in collagen scaffolds to 82 ± 12 μm and 75 ± 9 μm (*p* < 0.05) in those with 1% MWCNTs and 10% curcumin-MWCNTs 1%, respectively. This increased pore size enhanced hydroxyapatite crystal formation, surface wettability, *in vitro* biocompatibility with stem cells, and favorable *in vivo* biocompatibility in a rat model.

Moreover, freeze-drying is a gentle process that occurs under mild conditions, allowing for the incorporation of sensitive compounds and biological molecules, such as growth factors or proteins. Researchers have incorporated biphasic calcium phosphate (BCP), a mixture of hyaluronic acid and tricalcium phosphate that is widely used for bone regeneration, into porous CNT/polyvinyl alcohol (PVA)/BCP scaffolds (Lan et al., 2019). The elastic modulus of pure PVA hydrogels was measured at approximately 15 ± 1 kPa. The incorporation of BCP powder into PVA raised the elastic modulus to 48 ± 2 kPa. Introducing CNTs (at concentrations of 0.05% and 0.25%) along with 5% BCP particles further increased the elastic modulus of the PVA/BCP/CNT hydrogel from 53 ± 4 kPa to 81 ± 6 kPa. However, limitations to the freeze-drying technique remain. Compared to inkjet and extrusion-based printing, freeze-drying is more time-consuming (24–48 h) (Lan et al., 2019; Khoramgah et al., 2020). Hydrophobic polymers such as PDMS and ABS are not compatible with freeze drying, or require toxic organic solvents whose residue could harm cell viability (Fereshteh, 2018; Capuana et al., 2021).

Of the many avenues that exist to fabricate CNT-based scaffolds, 3D-printing is becoming increasingly relevant due to the high control researchers have over the final microstructure of the scaffold. Specifically, multi-material 3D printing is a new field of additive manufacturing in which organic, inorganic, and cellular materials can be extruded at once (Sodupe-Ortega et al., 2018; Diaz-Gomez et al., 2019). More research should focus on harnessing multi-material 3D printing to (i) construct a micro-patterned conduit system from a CNT-based composite, and (ii) bioprint a mixture of living progenitor cells into precise positions along the conduit system. Such a solution would allow for precise control of differentiation, proliferation, and electrical stimulation. Eventually, it may be possible to customize a 3D-printed CNT scaffold from diagnostic imaging and implant it *in vivo* to interface with native nerve architecture directly.

## 3 Applications and challenges of CNT-based scaffolds

### 3.1 CNT-based scaffolds in neural tissue engineering

**FIGURE 4:**
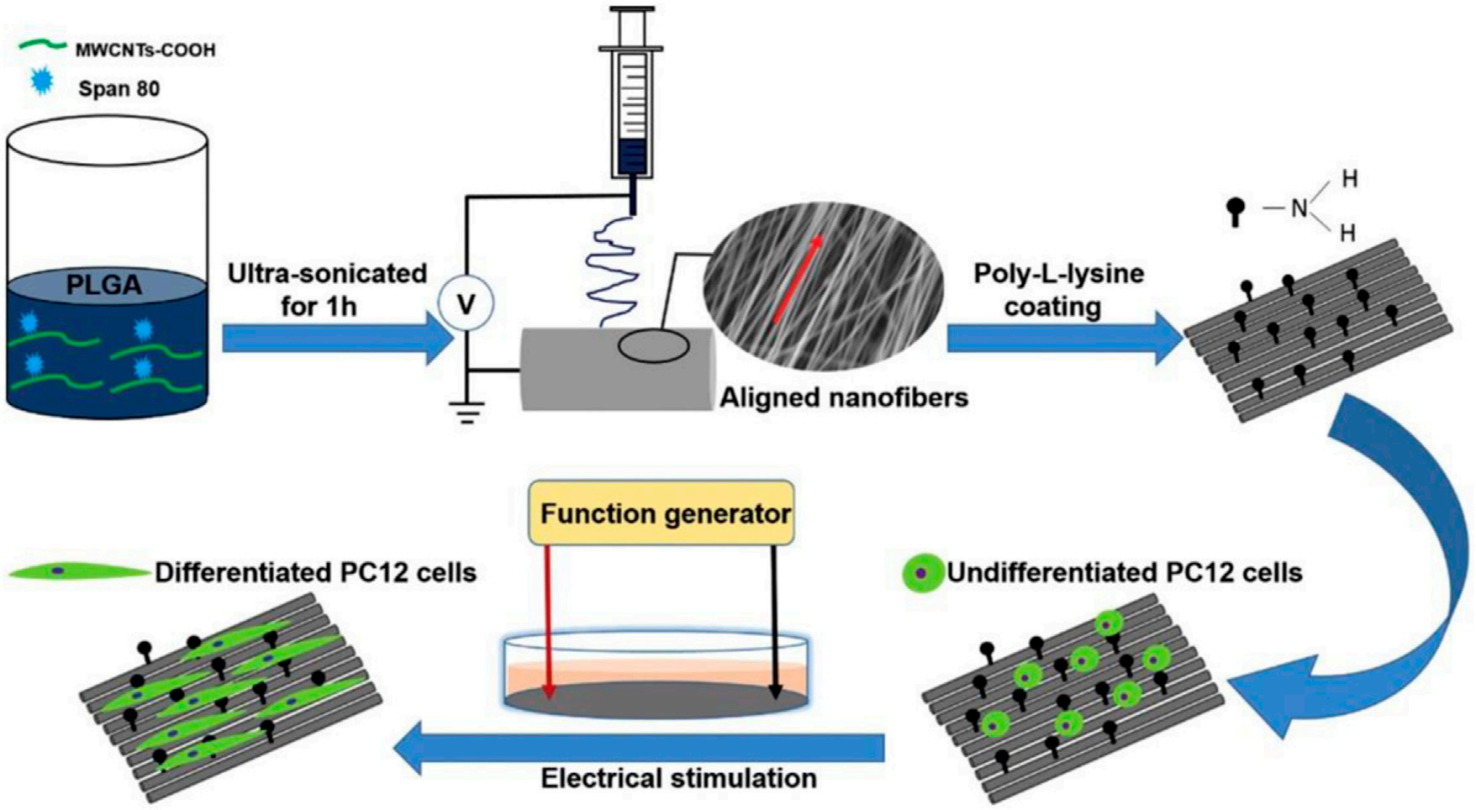
MWCNT was first ultrasonicated in a solution of PLGA in hexafluoroisopropanol (HFIP). Following electrospinning, the fibers were incubated in poly-L-lysine solution for 12 h. Electrical stimulation of the coated scaffolds induced differentiation of PC12 cells, as evaluated by immunostaining of NF200. Reprinted with permission from Wang et al. (2018). Copyright 2018, Elsevier.

Well-dispersed carbon nanotubes impart conductivity to scaffolds, making CNT-based scaffolds uniquely primed to address challenges in neural tissue engineering. Processes that are core to nervous regeneration, such as axon regeneration, cell body migration, and nerve cell adhesion, benefit from a conductive microenvironment (Burnstine-Townley et al., 2020; Halim et al., 2021). CNT-based scaffolds can accurately mimic the mechanical and electrical properties of the peripheral and central nervous system. Studies with HT-22 hippocampus neurons showed that an aligned MWCNT-chitosan composite had great biocompatibility. The direction of MWCNT alignment served as a template for the growth of hippocampus neurons, providing pathways for carefully regulated cell migration across a scaffold (Gupta et al., 2015). MTT assays showed high viabilities of 90%–120%, indicating strong biocompatibility of the scaffold. In a different study, poly-L-lysine was applied to PLGA-MWCNT to provide a hydrophilic surface for nerve cell adhesion (Figure 4). Electrical stimulation increased neuronal differentiation and Schwann cell myelination, and PC12 and dorsal root ganglion (DRG) neurons developed in the fiber direction (Wang et al., 2018). PC12 cells expressed NF200, a specific protein-neurofilament, on all nanofibrous scaffolds. In other studies, CNT may act not only as a conductive nanofiller, but also as a substrate for coatings of neuroactive drugs. Surface modification of PLGA/CNT nanocomposites with laminin, a neurite promoting protein, induced significantly longer neurite extensions than unmodified scaffolds (Nazeri et al., 2021). The mechanism for laminin-induced neurite outgrowth has been attributed to activation of FAK-MERK/ERK signaling pathways. Additionally, the laminin-modified scaffolds were biodegradable, losing 20% of total mass over 4 weeks of incubation.

**FIGURE 5:**
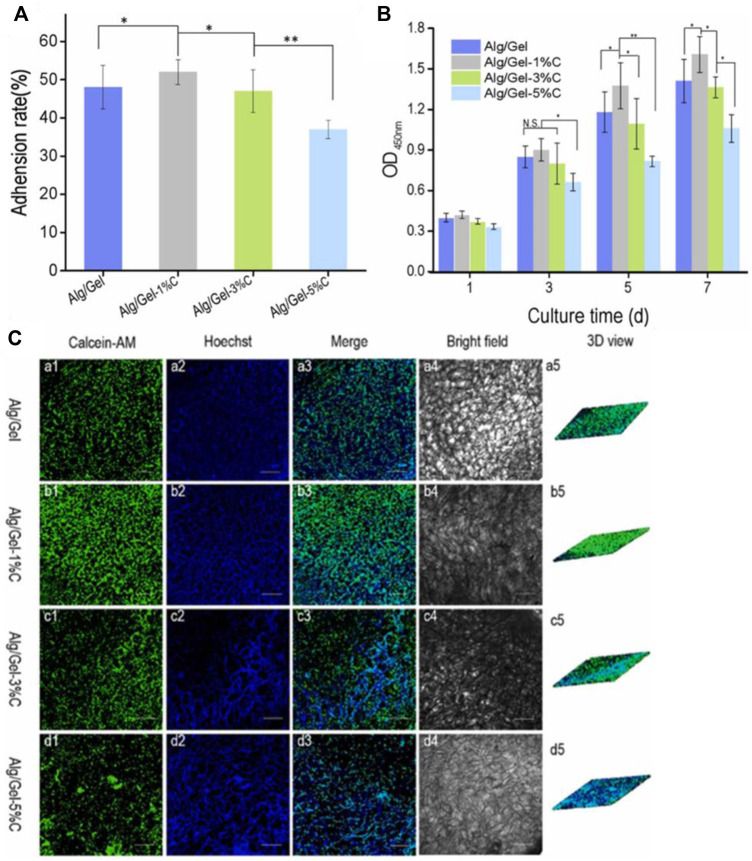
**(A)** Adhesion rates were highest for Alg/Gel-1% CNT scaffolds, indicating an intermediate loading of CNT is optimal for neural cell attachment and proliferation. **(B)** As obtained from CCK assays, optical density (OD) measurements indicate the highest cell proliferation in 1% CNT scaffolds, while 3% and 5% CNT scaffolds showed a small decrease in viability. **(C)** Spectroscopy measurements and 3D rendering of cellular activity reinforce that the highest cellular density is found in alginate/gelatin scaffolds with 1% CNT loading (images taken after 5 days of culture). Reprinted with permission from Ma et al. (2023). Copyright 2023, Elsevier.

Carbon nanotubes have been incorporated into polymer blends. Unlike CNT-nanocomposites formed from single polymers, polymer blends can be tuned to match the hydrophilicity and mechanical properties of the native tissue environment. For example, carboxyl-modified MWCNT was introduced to sodium Alg/Gel scaffolds (Ma et al., 2023). It was found that hydrophobicity, conductivity, and mechanical properties were improved, and PC12 cells proliferated over time (Figure 5). CCK-8 assays demonstrated high cell viability and even cell growth over 7 days. Moderate (1%) loadings of CNT were favored, while higher (3%, 5%) CNT loadings decreased porosity and cell adhesion rate. At a CNT concentration of 1%, the scaffolds exhibited a surface contact angle of ∼72°, porosity of 90%, compression modulus of ∼0.7 MPa, and conductivity of ∼5 × 10^−4^ S/m, all of which are close to the native growth environment for PC12 cells. Importantly, whisker-like neurite structures were observed on the Alg/Gel/MWCNT scaffolds, indicating intercellular contact. Such intercellular communication is key to synaptogenesis and signal transduction, strengthening the potential of such composites as nerve tissue engineering scaffolds. In this study, the presence of CNT was enough to promote neural cell growth by mimicking the contact angle, porosity, compression modulus, and conductivity of the neural environment. In addition to incorporating CNT into scaffolds, researchers have studied electrical stimulation as a way to mimic the neural ECM even more closely, by imitating action potentials used for neural communication.

Electromagnetic (EM) stimulation, the application of a potential difference, magnetic field, or both, has been explored as a way to further increase cellular response and regrowth in neural tissue engineering systems. The mechanism of this action has been linked to the endogenous production of an electric field at a wound during the normal healing process. Passing an electric field through a scaffold simulates the wound healing process and enhances nerve growth and sprouting (McCaig et al., 2005). Specifically, electric fields increase mitochondrial metabolism, intracellular calcium, and actin redistribution, all of which are processes key to neuronal migration and plasticity (Zhu et al., 2019). The standard for electrical stimulation procedures involves electrical pulses of 20 Hz for 1 h per day (Li et al., 2020c; Javeed et al., 2021). Conducting electrical stimulation longer than 1 h reduces gains in sensory neuron regeneration. It appears that longer periods of stimulation over-sensitize and decouple the tyrosine kinase b (trkB) receptor, a key receptor in neuronal differentiation and survival. Electrical currents greater than ∼4 mA provide excessive direct current and inhibit fiber growth. Meanwhile, electrical currents of ∼1 mA provide more mature regeneration of nerve architecture, with higher myelination, vascularity, and a smaller cross-sectional area of regenerated axons.

In another study, electrospun poly (p-dioxanone) (PPDO)/CNT nanoyarns were electrically stimulated to enhance the differentiation of human adipose-derived mesenchymal stem cells (hADMSCs) into Schwann-like cells (Wu et al., 2022). When CNT was introduced, the nanoyarns assumed a rough topography. These geometric patterns accelerated the contact guidance phenomenon, providing a high surface area environment that led to improved biocompatibility and phenotypic maintenance of the Schwann-like cells. Further, researchers observed robust expression of neurotrophic factors, such as nerve growth factor (NGF) and hepatocyte growth factor (HGF). Vascular endothelial growth factor-A (VEGF-A) was heightened in all experimental groups, demonstrating potential for angiogenesis and nerve regeneration. In the previously mentioned study on Alg/Gel/MWCNT scaffolds, magnetic fields elongated neurite structures of PC12 cells, with maximum effects reached at ∼ 1 mT and ∼50 Hz (Ma et al., 2023). Commonly used magnetic field parameters range from ∼0.1–3 mT and ∼15–75 Hz, indicating an intermediate parameter may be optimal. The effect of magnetic fields on PC12 neurogenesis was attributed to the upregulation of the extracellular signal-regulated kinase 1/2 (ERK-1/2) signaling pathway involved in cell division. This pathway serves as a crucial conduit for transmitting extracellular signals to the nucleus, orchestrating a finely tuned symphony that governs the cell cycle, cellular proliferation, and developmental processes.

As nerve regeneration scaffold technology advances, trends have shifted to designing advanced nerve guidance conduits (NGCs) with complex internal structures to maximize axon regeneration rate and avoid muscle atrophy. Specifically, the use of multiple materials is being explored, in which the outer layer is composed of a CNT-based electrospun composite, while the internal filler is composed of an injectable hydrogel or a porous matrix (Madhusudanan et al., 2020). The conduits are designed such that the external layer would be mechanically similar to a human nerve (∼6.5 MPa < ultimate tensile strength < ∼ 8.5 MPa), and the internal filler would be of optimal porosity (∼60–80% porosity degree, pore size ∼30–50 μm) for axon growth (Chiono and Tonda-Turo, 2015). If an NGC with the above listed features shows improved performance, such scaffold may 1 day replace autologous grafts as the gold standard approach to nerve regeneration.

### 3.2 CNT-based scaffolds in cardiac and muscle tissue engineering

The potential of CNT-based scaffolds also extends into cardiovascular tissue engineering. Besides mimicking the naturally conductive microenvironment of cardiovascular tissue, aligned CNT scaffolds generate narrowed cardiomyocyte morphologies and anisotropic organization. One study fabricated nanofiber scaffolds using PVA, chitosan, and varying concentrations of CNTs through electrospinning (Mombini et al., 2019). Analyses encompassing scanning electron microscopy (SEM), mechanical testing, electrical conductivity, water uptake, cell adhesion, and viability, showed that a concentration of ∼1% was optimal for cardiac differentiation. Furthermore, in investigations of differentiation of rat mesenchymal stem cells (MSCs) into cardiomyocytes through electrical stimulation, real-time qPCR results exhibited a significant (>∼ 3-fold) upregulation in the expression of Nkx2.5, Troponin I, and β-MHC cardiac markers compared to the control group. Overall, these findings suggest that the integration of MSCs, conductive scaffolds, and electrical stimulation holds substantial promise as a valuable approach in cardiac tissue engineering.

It is crucial that scaffolds for cardiovascular tissue engineering possess similar mechanical characteristics and are not susceptible to the development of irreversible creep when the structure is exposed to blood pressure. This requires precise control of polymeric mechanical properties. Due to the high Young’s modulus of CNT, coupled with ease of dispersion, they are optimal for this application as highly tunable, mechanically enhancing nanofillers. One study employed electrospinning of Gel/SWCNT/polyurethane nanofibers as a solution (Tondnevis et al., 2020). The composite scaffolds exhibit biomimetic mechanical properties resembling normal blood vessels, with SWNTs enhancing the Young’s modulus and ultimate strength. Conversely, the addition of gelatin increases elongation at break due to its softening effect. Biological evaluation using SEM and MTT assay reveals the formation of a dense layer of myocardial myoblast and endothelial cells on the nanofibrous surface after 7 days of culture, indicating an avenue towards regenerating cardiac tissue of multiple cell types.

**FIGURE 6:**
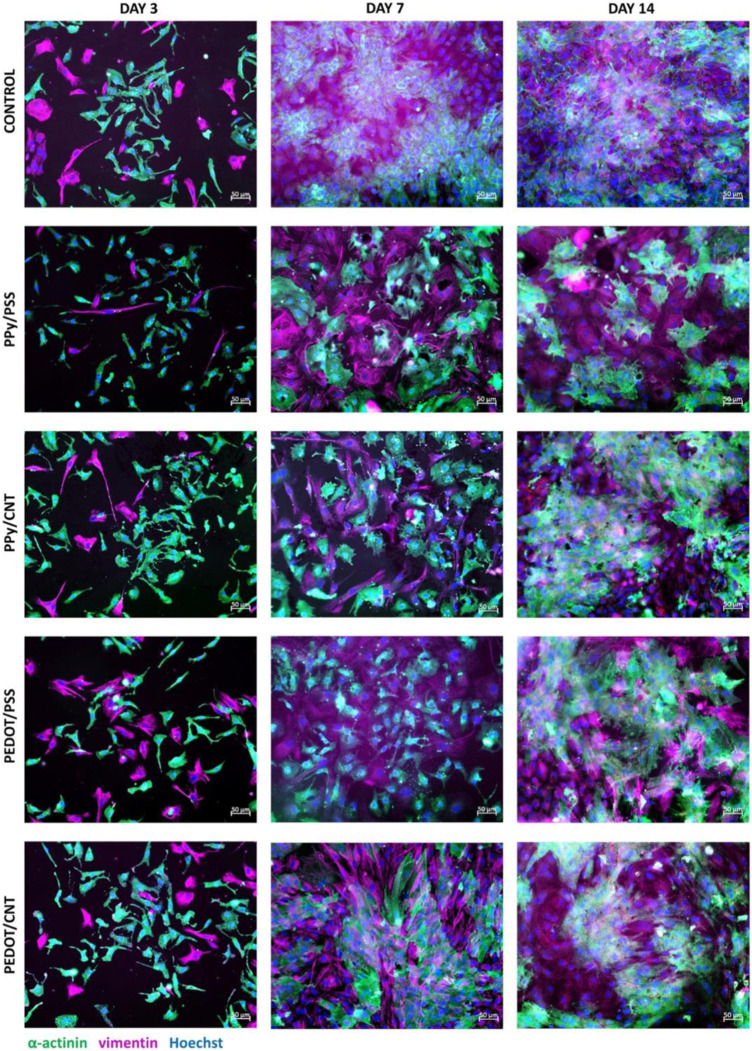
Comparison of cellular imaging of control, PPy/PSS, PPy/CNT, PEDOT/PSS, and PEDOT/CNT scaffolds after 3, 7, and 14 days of culture. Immunostaining revealed that after 7 and 14 days of culture, CNT-based substrates supported the growth of thinner and smaller fibroblasts, consistent with a non-pathological phenotype. Thus, CNT-based substrates may promote better proliferation of cardiomyocytes by controlling fibroblast growth. Reprinted with permission from Alegret et al. (2022). Copyright 2022, Springer Nature.

CNT-based scaffolds hold remarkable promise as a tool to promote the interface between cardiovascular and muscle tissue engineering. One study investigated two different blends of CNT with conductive polymers, polypyrrole (PPy)/CNT and poly (3,4-ethylenedioxythiophene) (PEDOT)/CNT, to assess their impact on the growth, survival, and beating behavior of neonatal rat ventricular myocytes (NRVM) (Alegret et al., 2022). NRVMs cultured on CNT-based substrates showed enhanced cardiac functionality, characterized by homogeneous, non-arrhythmogenic, and more frequent spontaneous beating, with PEDOT/CNT substrates producing higher beating amplitudes indicative of a more mature cardiac phenotype (Figure 6). Furthermore, the cells exhibit enhanced structural features such as aligned sarcomeres and abundant Connexin 43 (Cx43) organization. Importantly, no signs of induced hypertrophy were observed. Hypertrophy in cardiac muscle tissue, or abnormal thickening of tissue, reduces contractility and in extreme cases, leads to cellular death (Tani and Tohyama, 2022). It was also found that after 7 and 14 days of culture, substrates with carbon nanotubes produced fibroblasts with slender morphologies and non-activated natures. Fibroblasts are key players in regulating ECM synthesis and deposition, intercellular communication, and cardiomyocyte electrical activity. As CNT scaffolds play a role in controlling fibroblast proliferation and promoting a non-pathological nature, they can enhance cardiomyocyte survival.

**FIGURE 7:**
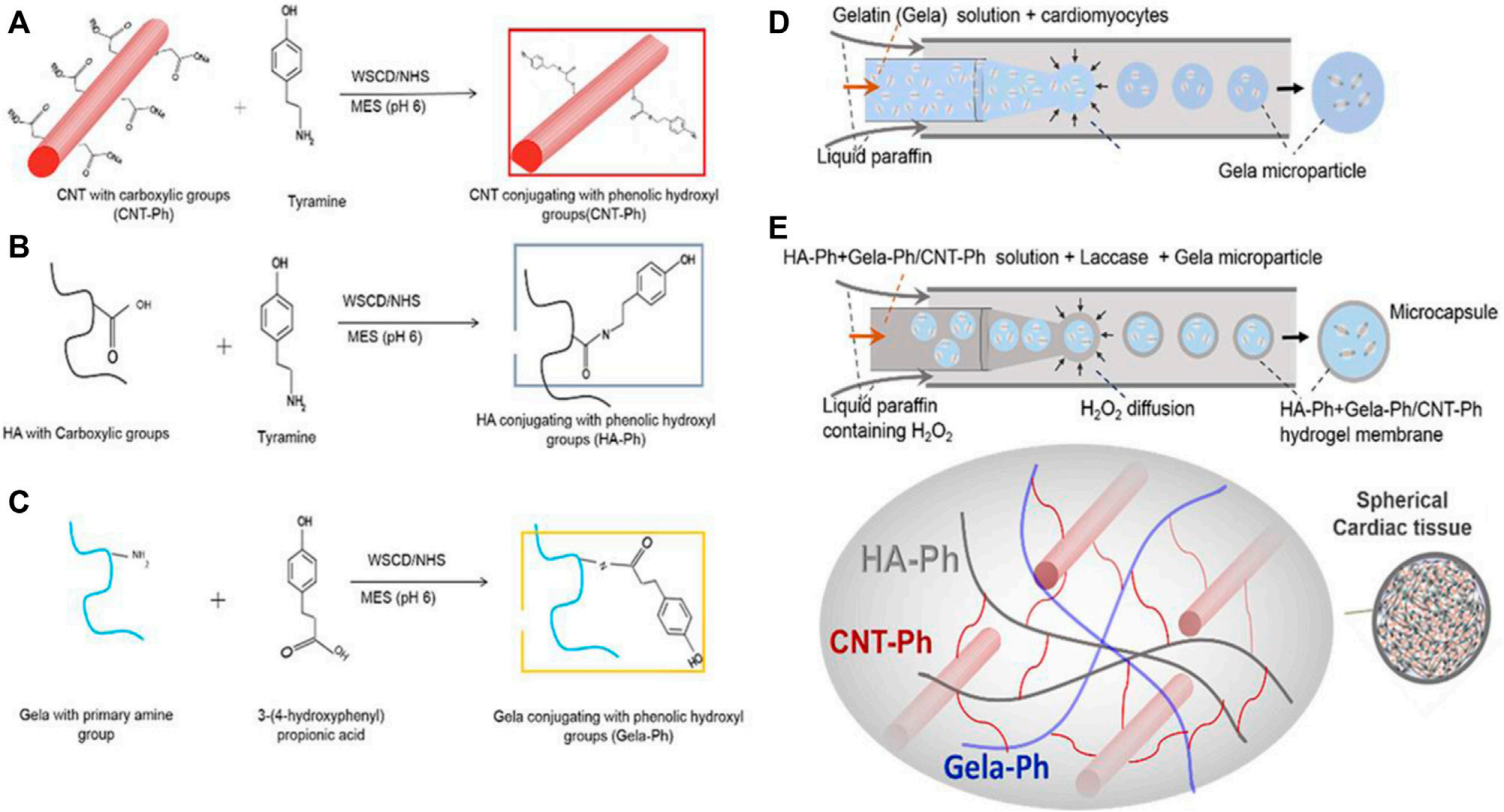
**(A–C)** Conjugations of CNT, HA, and gelatin with phenolic hydroxyl groups provide a template for chemical crosslinking to occur. **(D)** Cardiomyocytes are packaged into gelatin microparticles. **(E)** With the assistance of the laccase enzyme, gelatin microparticles are encapsulated with crosslinked HA-Ph/CNT-Ph/Gel-Ph. No significant decrease in cellular viability is observed from the encapsulation process. Reprinted with permission from Sharifisistani et al. (2022). Copyright 2022, Wiley-VCH.

As researchers look for future directions, novel techniques to incorporate desirable electrical and mechanical properties of CNT are being developed with applications in cardiac tissue engineering. A recent study introduced a microfluidic-based encapsulation process for fabricating biomimetic hydrogel microcapsules (Sharifisistani et al., 2022). Phenol-substituted gelatin (Gel-Ph) and CNT (CNT-Ph) were incorporated into a HA-based hydrogel through laccase-mediated crosslinking, serving as cell-adhesive and electrically conductive substrates (Figure 7). The encapsulation process does not adversely affect cellular viability, and the cells harvested from the microcapsules proliferate similarly to subcultured cells on tissue culture plates. The designed hydrogel, covalently decorated with Gel-Ph and CNT-Ph, exhibited significantly improved biophysical properties, including mechanical strength, swelling, biodegradability, and electroconductivity. Spheroid formation of cardiomyocytes within the microcapsules was observed 3 days after encapsulation. Moreover, the encapsulated cardiomyocytes exhibited increased expression of cardiac markers, such as the Actn-4 and Conx43 proteins, involved in cytoskeletal and gap junction activities.

Recent years have seen large advances in the application of CNT scaffolds for cardiac and muscle tissue engineering. However, there is room for improvement, such as the use of machine learning in designing micro-physiological systems. Machine learning would allow for high-throughput analysis and fabrication of scaffold geometry and cellular response, enabling researchers to efficiently maximize survival (Nguyen et al., 2019). Moreover, researchers should examine the possibility of genome editing as a tool to improve the immune resistance and robustness of implanted cardiomyocyte cells. Specifically, cardiomyocytes may be genetically modified to better withstand cryopreservation, allowing for more robust cells to be implanted into the final CNT scaffold (Sharma et al., 2021).

### 3.3 CNT-based scaffolds in bone tissue engineering

**FIGURE 8:**
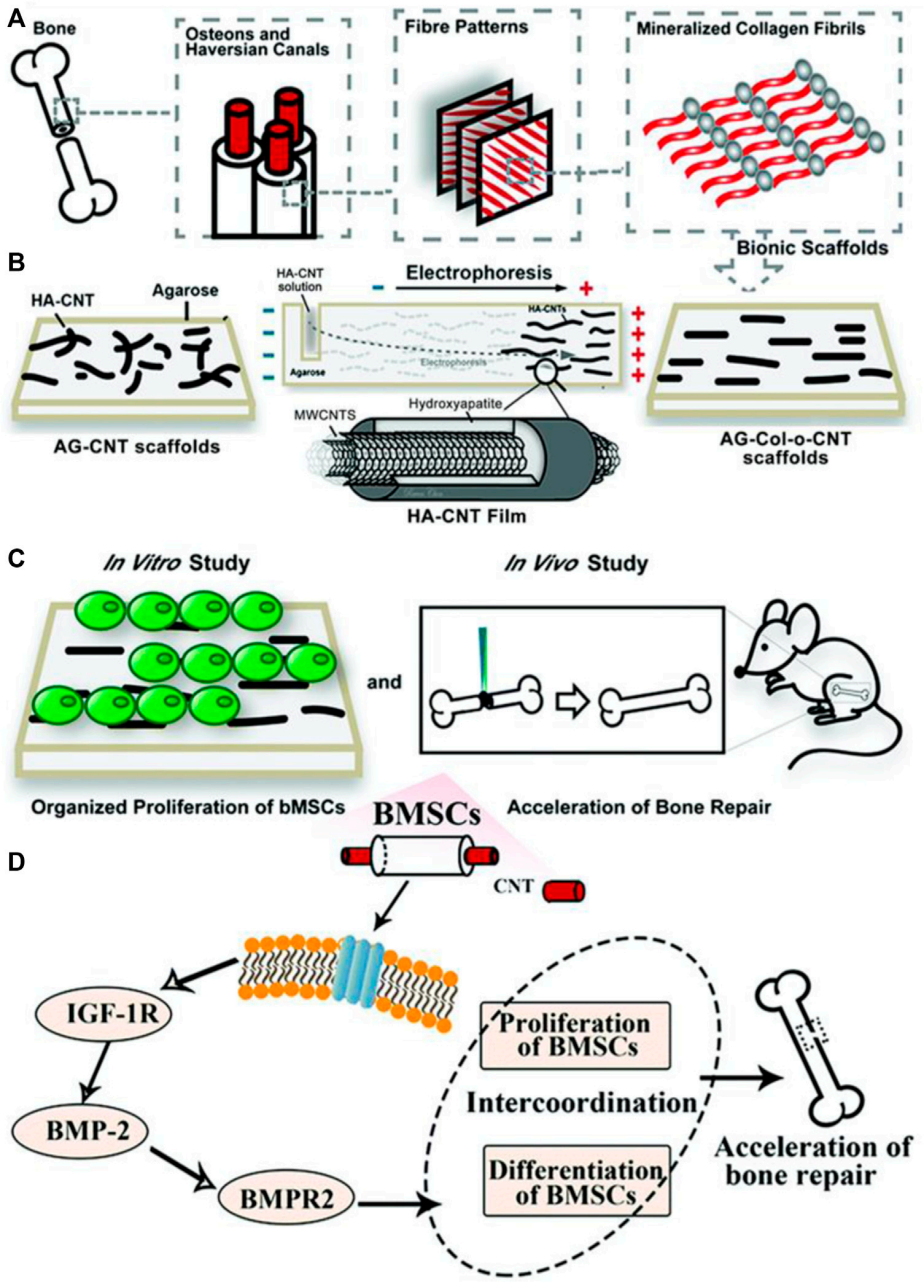
**(A,B)** To mimic the fiber patterns in normal bone structure, researchers employed an AG electrophoresis setup. HA-CNT was loaded into one end of an AG gel and subjected to a potential difference, causing it to migrate towards the anode, creating a striated pattern. **(C)** *In vitro* proliferation of bMSCs was studied and demonstrated coordination between proliferation and differentiation of bMSCs. *In vivo* studies with rat models showed transplantation of scaffolds into bone defects effectively repaired them within 56 days. **(D)** The scaffolds facilitated the proliferation of BMSCs and additionally impacted the transformation of BMSCs into osteoblasts. Reprinted with permission from Liu et al. (2020a). Copyright 2020, Royal Society of Chemistry.

Bone tissue regeneration relies extensively on a sound mechanical environment to promote cell growth. One way to produce scaffolds with enhanced mechanical properties is through the addition of carbon nanotubes. The use of a carbon nanotube-hydroxyapatite composite has shown promising results in cell growth promotion. One study aimed to create ordered CNT-HA scaffolds by utilizing AG hydrogel electrophoresis to mimic the biomimetic parallel pattern observed in Col and hydroxyapatite hydrogel scaffolds (AG-Col-o-CNT) (Liu et al., 2020a). This property of CNT to form biomimetic ordered parallel structures is not shared by 1D- or 3D-conductive nanofillers. Thus, CNT are uniquely positioned as versatile inducers of osteoregeneration. *In vitro* experiments revealed that the AG-Col-o-CNT scaffolds accelerated the proliferation and differentiation of bone mesenchymal stem cell lines (Figure 8).

Furthermore, when transplanted into bone defects after 28 and 56 days, the scaffolds effectively repaired the defects in rat models. After 28 days, the size of bone defects significantly decreased in AG-o-CNT and AG-Col-o-CNT groups compared to the control AG group. After 58 days, there were obvious periosteal lines and dense bone structure in the AG-o-CNT and AG-Col-o-CNT groups as well. Collagen fibers were smoother in the latter. Masson staining further showed that the pure agarose scaffold group showed lower density of collagen type 1 at the bone defects than the scaffolds with CNT. Finally, white blood cell and lymphocyte counts were not significantly different in experimental groups as compared to control groups after 5 days, indicating no toxic side effects from the MWCNT treatment. Such *in vivo* studies are promising evidence that CNT-based scaffolds are powerful biocompatible and osteoregenerative tools.

Amine-functionalized SWCNTs were incorporated into PCL in varying concentrations (0%–0.5%) to enhance the structural and functional properties of electrospun scaffolds (Tohidlou et al., 2019). The attachment, proliferation, and differentiation of rat bone marrow-derived mesenchymal stem cells (rMSCs) seeded onto the scaffolds were analyzed. While the scaffold with moderate SWCNT loading (∼0.2 wt%) demonstrated optimal mechanical properties, the composite scaffold with ∼0.5 wt% SWCNT demonstrated superior proliferation and differentiation of rMSCs. Increased alkaline phosphatase activity indicates elevated differentiation of cells on the nanocomposite scaffolds. These positive changes are attributed to two key effects. The first is that functional groups regulate protein adhesion and integrin binding; amino and hydroxyl groups have been shown to best promote this activity. The second factor may be due to surface roughness, which changes cell-matrix adhesions and signaling pathways. Unlike neural tissue engineering, electrical stimulation is less common in bone tissue engineering. While neurites and glia rely on an electrical environment to survive, the main function of osteocytes is to secrete proteins and maintain the mineral matrix of bone tissue. Therefore, CNT’s effects on protein adhesion, surface roughness, and mechanical strength outweigh gains from electrical conductivity.

**FIGURE 9:**
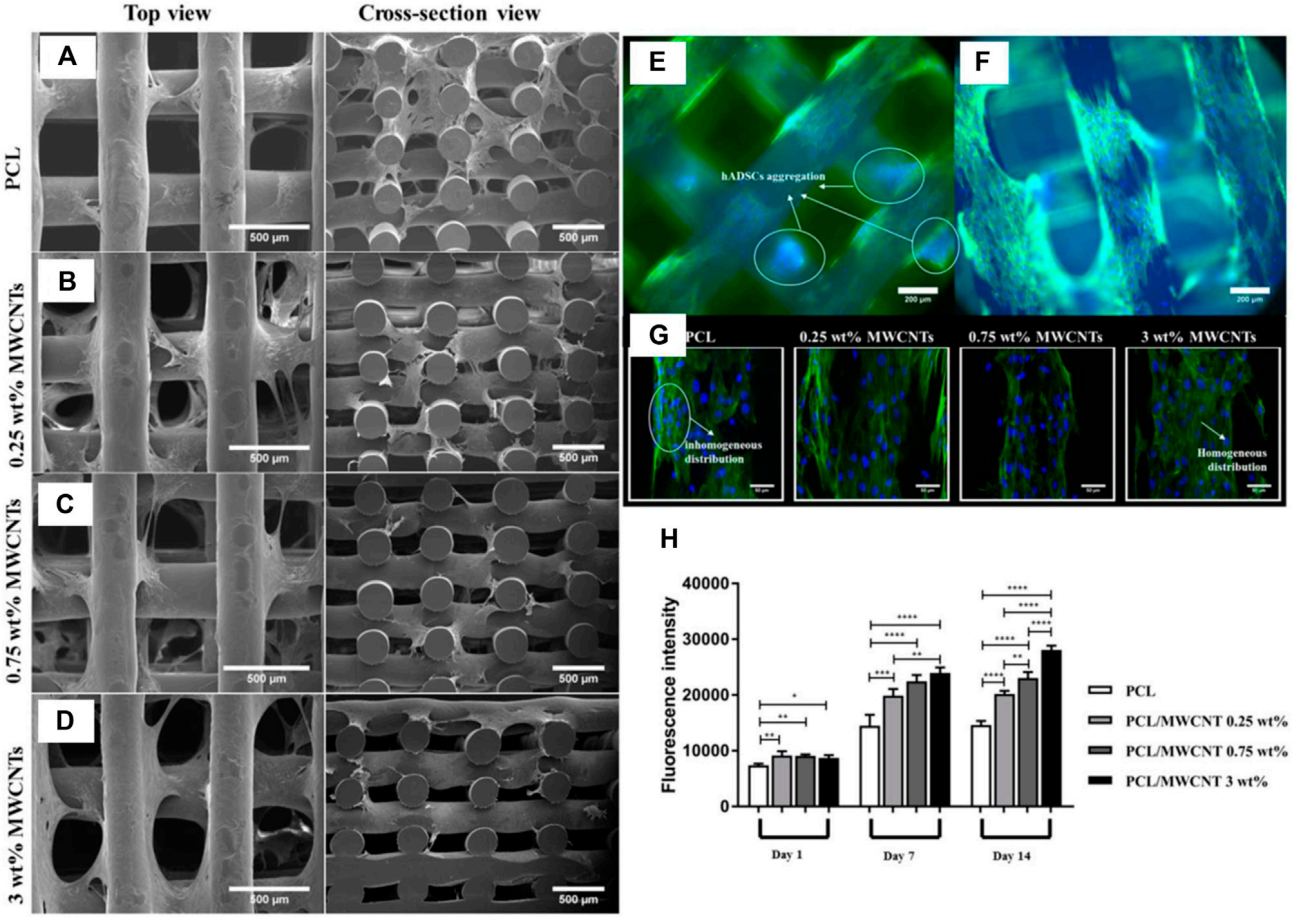
**(A–D)** Using screw-assisted extrusion-based additive manufacturing, researchers constructed porous scaffolds of PCL/MWCNT. **(E,F)** Fluorescence microscopy images of PCL and 3% PCL/MWCNT show a decrease in hADMSC aggregation in the latter scaffold. **(G)** Confocal microscopy further reveals homogenous distribution in PCL/MWCNT scaffolds compared to pure PCL scaffolds, indicating the electrical properties of CNT enhances cellular communication and uniform distribution. **(H)** Alamar blue results demonstrate a direct relationship between % CNT loading and cellular proliferation levels. Influence of CNT on cellular activity is enhanced on days 7 and 14. Reprinted with permission from Huang et al. (2019). Copyright 2019, Elsevier.

3D-printing techniques have been applied to CNT scaffolds to form 3D porous scaffolds with fully tunable topologies. Porosity of scaffolds is important as it determines cell infiltration, vascularization, migration, and the rate of nutrient flow (Chen and Kawazoe, 2018; Abbasi et al., 2020). Pores provide a concave surface that provides room for cell alignment, decreases cell stress, and reduces cell surface energy. Moreover, a larger pore size increases scaffold degradation rate, while ultra-high molecular weight scaffolds with smaller pore sizes will degrade slowly and be of limited use. Studies suggest that pores of ∼250–400 μm are the optimal size for macrophages to eliminate pathogens while also allowing cells to colonize and migrate *in vivo*. In one example, PCL/MWCNTs scaffolds with varying concentrations of MWCNTs (0.25, 0.75, and 3 wt%) were successfully fabricated using screw-assisted extrusion-based additive manufacturing (Huang et al., 2019). Pore sizes in the study ranged from ∼366–397 μm. At ∼ 3 wt% MWCNT, the compressive modulus was closest to cancellous bone. Interestingly, it was observed that increasing MWCNT concentration leads to aggregation of small-sized MWCNTs due to strong van der Waals interactions, which enhances the interface area with cells and improves cell attachment and protein adsorption (Figure 9). However, controlling this aggregation is crucial to prevent the formation of large aggregates that may hinder cell-cell interactions. No cytotoxic effects are observed due to the presence of MWCNTs, and the scaffolds were able to support early-stage human adipose-derived mesenchymal stem cells proliferation.

Among the newest advances in bone tissue engineering is the development of smart scaffold materials that enable the quantification of new bone formation. One study integrated carboxylated CNTs into chemically cross-linked carboxymethyl chitosan hydrogel to create a noninvasive and intelligent monitoring scaffold for bone regeneration (Huang et al., 2023). To improve osteogenic differentiation, bone morphogenetic protein 2 (BMP2) was added as a growth factor. Voltammetry and impedance studies were used to identify the osteogenic potential of CNT composite scaffolds, and rigorous biological evaluations were conducted to confirm this potential. Specifically, researchers studied the electrochemical signal response of CNT/carboxymethyl chitosan (CMC) substrates during osteogenic differentiation *in vitro*. As the mineralization matrix deposited, the cyclic voltammetry (CV) curve showed a low peak current value because the charge transfer ability of the bone cells decreased with cell differentiation time. Moreover, conductivity of the CNT/CMC scaffold decreased after 2 weeks of cell culturing as the matrix became more mineralized and CNT% slowly decreased. Through these results, researchers were able to confirm that electrochemical responses could be used as an indirect way to monitor progress bone regeneration. Notably, the CNT scaffold overcomes the deactivation issue of bone morphogenetic protein 2 by sustainably enhancing stem cell osteogenic differentiation in both *in vitro* and *in vivo* studies. In fact, picrofuchsin staining of collagen fibers was conducted to examine mineralization degree in new tissues, showing that CNT/CMC/BMP2 nearly filled the gap with collagen fibers *in vivo*. This introduction of a bioactive CNT composite scaffold could be key for long-term, non-invasive monitoring of bone repair.

Further research in bone tissue engineering should focus on incorporation of not only CNT but also other materials in novel nanocomposites. Bioactive glass and metallic materials are widely used due to high bioactivity, bone binding abilities, and ability to provide mechanical support (Dec et al., 2022). Often, materials such as titanium (Ti) and Ti-based alloys have been used in orthopedic implants, but the inertness of Ti can lead to formation of fibrous tissue (Tang et al., 2021). Moreover, there is a mechanical mismatch between Ti and bone tissue that could hinder bone formation and absorption. Future directions in bone tissue engineering research should focus on CNT-metal composites that could provide the best of both worlds. An advantage of CNT is the ability to tune mechanical properties through CNT% loading, as well as improved protein adhesion. Harnessing these advantages, along with the biocompatibility of Ti, could lead to next-generation bone implants capable of stabilizing bone injury sites and accelerating bone regeneration.

### 3.4 Cytotoxicity of CNT-based scaffolds in tissue engineering

An important topic in the viability of CNT scaffolds in tissue engineering is cytotoxicity. Length of CNT is a large factor affecting toxicity of CNTs. Shorter CNTs (length <1 μm) are found to pass through the cell membrane more easily, and lead to negative effects. These detrimental effects include oxidative stress, DNA damage, cell membrane injury, amino acid oxidation, and apoptosis. It seems that increase in reactive oxygen species (ROS) is the primary pathway through which CNT accumulation induces oxidative stress, which in turn leads to damage in genetic material and alterations in the cell cycle (Cheng et al., 2011; Azad et al., 2013). Specifically, SWCNT leads to hydroxyl radical production, activating activator protein-1 (AP-1) pathways, mitogen activated protein kinase (MAPK) pathways, and nuclear factor-kB (NF-kB) pathways of inflammatory response (Sabido et al., 2020). This effect is reduced for MWCNTs, as the greater delocalization area increases MWCNT’s scavenging capacity. Its ability to scavenge toxic free-radicals leads to less oxidative stress and fibrosis than SWCNT. Because MWCNT are both less cytotoxic and more accessible compared to SWCNT, they may be better suited for CNT tissue engineering applications.

In a study on MWCNT-hydroxyapatite scaffolds, MTT test investigations on *in vitro* cell viability and proliferation showed that integrated MWCNTs did not have a significant cytotoxic effect on the cells when incubated for 24 and 48 h (Ravoor and Renold Elsen, 2022). Moreover, degradation experiments showed stability of the scaffolds in PBS for 35 days. During the incubation period, the scaffolds’ weight increased due to apatite layer formation. It is believed that MWCNTs may serve as nucleation sites for apatite formation, providing areas vital for osteoblast attachment (Akasaka et al., 2006). Such results suggest that while caution must be taken to ensure CNT loadings in bone regeneration scaffolds are not excessive, CNT-based scaffolds are biocompatible and osteoprotective on the whole.

**FIGURE 10:**
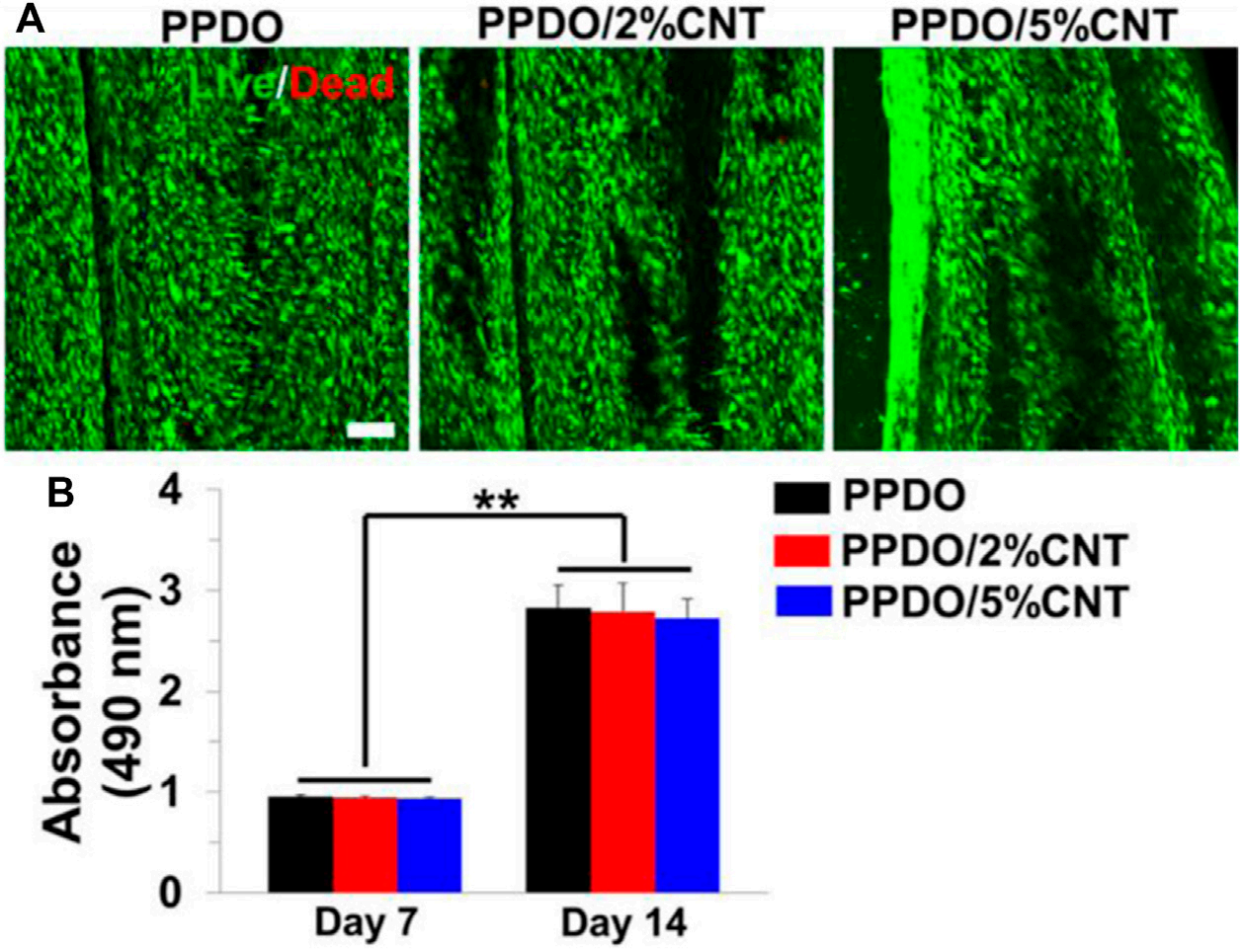
**(A)** Electrospun PPDO/CNT nanoyarns were tested at CNT concentrations of 2% and 5%. Live/Dead staining showed minimal cytotoxicity for both 2% and 5% CNT. Scale bar = 100 μm. **(B)** No significant differences in cell density were observed between the three groups on days 7 and 14. Reprinted with permission from Wu et al. (2022). Copyright 2022, Elsevier.

Studies on CNT scaffolds have focused on incorporating optimal concentrations of CNT that limit cytotoxicity while maintaining positive impact on mechanical and electrical properties. A neural engineering study on poly (p-dioxanone) (PPDO)/CNT nanoyarns utilized CNT concentrations of 2% and 5% (Wu et al., 2022) (Figure 10). Researchers also found that higher concentrations negatively affected the electrospinning process as it becomes difficult to homogenously disperse the CNT. Nevertheless, both ∼2% and ∼5% CNT loadings exhibited no cytotoxic effects, but instead provided a nanofibrous topography with high surface area. Thus, through the contact guidance phenomenon, the CNT scaffolds regulated cell morphology. Several studies use even lower concentrations of CNT in an effort to reduce cytotoxicity. MWCNT-PEGDA scaffolds that were 3D-printed for nerve regeneration used CNT concentrations of 0.02%, 0.05%, and 0.1% (Lee et al., 2018). Despite these lower concentrations, significant increases in Young’s modulus, average neurite length, and average length of longest neurite were still observed. Further, neuronal markers were elevated in MWCNT-PEGDA scaffolds. Because the increase in neuronal markers was promoted by electrical stimulation, it can be assumed that scaffolds with low concentrations of ∼0.02–0.1% CNT are still sufficiently conductive to transfer electrical charge uniformly.

**FIGURE 11:**
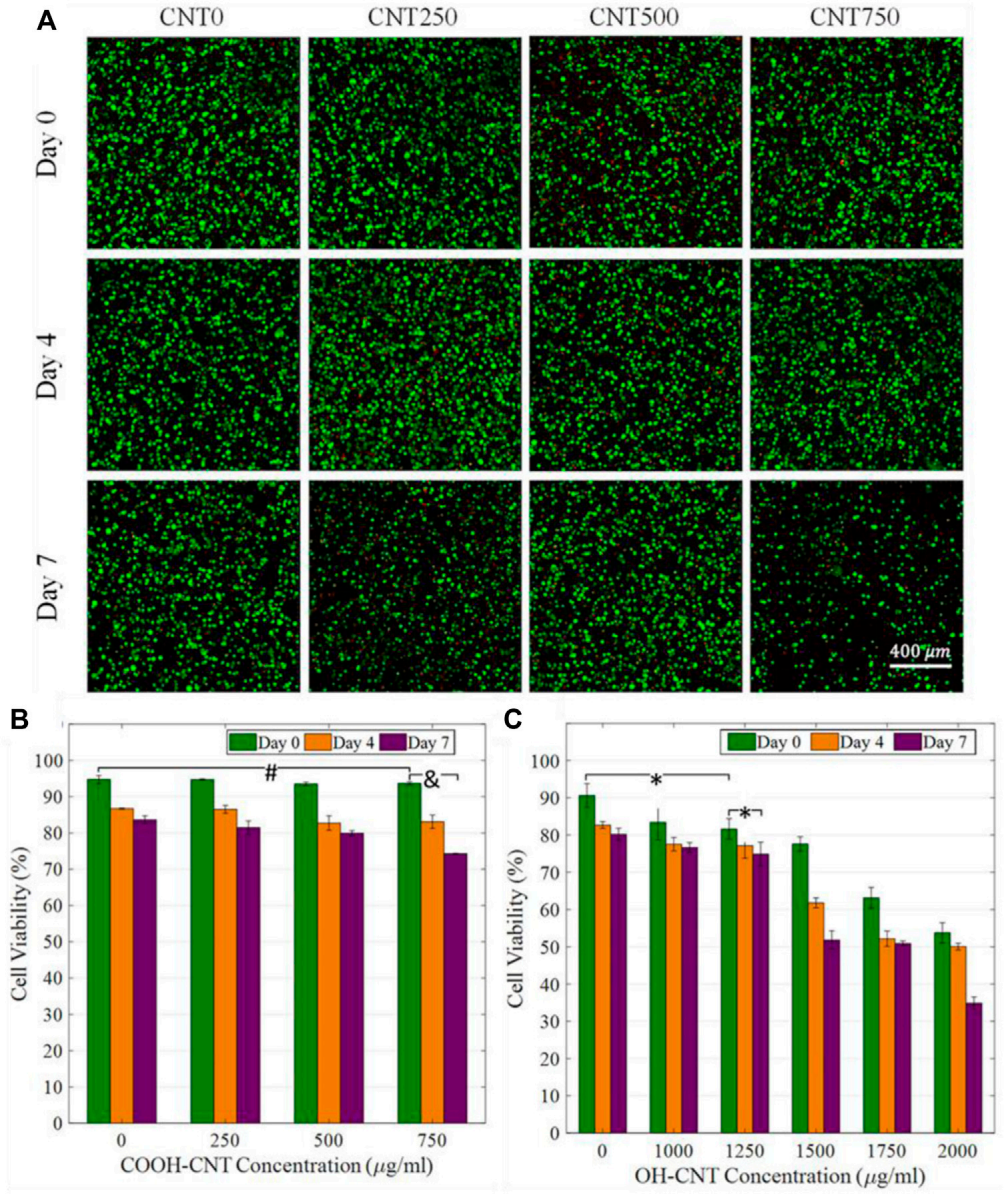
**(A)** Cell viability studies for CNT-COOH concentrations of 0, 250, 500, and 750 μg/mL. Small decreases in cell viability are observed. **(B,C)** Comparison of CNT-COOH and CNT-OH biocompatibility shows a higher critical concentration of CNT-OH is needed to exhibit similar cytotoxic effects as CNT-COOH. Reprinted with permission from Ravanbakhsh et al. (2019). Copyright 2019, Elsevier.

Other studies investigated the effect that functionalized CNT may have on the pH of the cellular microenvironment and how this may affect biocompatibility. Carboxylated CNT (CNT-COOH) and hydroxylated CNT (CNT-OH) add acidic and basic groups to CNT nanocomposites, respectively. A study examined differences in cellular response to glycol chitosan hydrogels with CNT-COOH and CNT-OH (Ravanbakhsh et al., 2019). The control glycol chitosan hydrogel exhibited a pH of ∼7.2; CNT-OH addition raised the pH near the normal physiologic pH range of ∼7.4–7.5, while CNT-COOH addition decreased the pH away from normal physiologic pH (Figure 11). pH affects virtually all cellular activities, from membrane potential to metabolism to polymerization of cytoskeleton. Consistent with this theory, researchers found CNT-OH was less cytotoxic and could be present in greater concentrations (∼1,000 μg/mL *versus* ∼500 μg/mL) than CNT-COOH to achieve the same cell viability. These results highlight the importance of choosing proper chemical groups to covalently functionalize CNT so as not to decrease biocompatibility.

As CNT is an extensively π-conjugated compound with no naturally occurring variants in nature, pristine CNT is not easily biodegradable. The *in vivo* fate of CNT has been studied to determine the extent of CNT accumulation and biodistribution. A purified DNA-encapsulated SWCNT complex was administered intravenously in mice; the nanotubes predominantly localized to the liver but persisted in other organs for up to 5 months (Galassi et al., 2020). Despite their long-term presence, the nanotubes exhibited high biocompatibility for up to 4 months, with minor changes in renal markers at 5 months, indicating suitability for preclinical applications. The *in vivo* kinetics of SWCNTs implanted in mice have also been observed through near-infrared fluorescence imaging (Hirata et al., 2019). SWCNT was implanted between the periosteum and parietal bone. Over 24 h, fluorescence in the cranial region gradually decreased, and no fluorescence was observed in other organs during whole-body imaging experiments. Even after 56 days, fluorescence persisted at the implantation site, with no detectable accumulation in other organs, supporting their potential as biomaterials. Both studies highlight the importance of considering factors such as nanotube type, purity, functionalization, and route of administration in assessing the biodistribution and biocompatibility of CNTs for diverse applications, including drug development and tissue engineering.

Nevertheless, to some extent, CNT has been shown to be biodegradable by macrophages (Elgrabli et al., 2015; Hou et al., 2016; Yang and Zhang, 2019). The biodegradation mechanism involves the activation of nicotinamide adenine dinucleotide phosphate (NADPH) oxidase during phagocytosis. This activation leads to an increase in oxygen consumption and the generation of reactive oxygen species (ROS), including hydrogen peroxide (H_2_O_2_), superoxide anion (O2⋅^−^), and hydroxyl radical (OH·). The respiratory burst, or oxidative burst, induced by ROS is responsible for degrading internalized particles or bacteria. In the case of CNTs, this oxidative burst is believed to be the primary mechanism for their degradation within macrophages (Yang et al., 2019). Experiments using ROS inhibitors and myeloperoxidase (MPO), as well as tests on NADPH oxidase-deficient mice, consistently showed that NADPH oxidase-dependent ROS generation is crucial for the biodegradation of CNTs by macrophages (Ding et al., 2016). The biological pathway involves the formation of O2⋅^−^, its conversion to H_2_O_2_ or reaction with nitric oxide (NO·) to generate ONOO-, and subsequent attack on CNT defects and unsaturated carbon bonds, resulting in the degradation of CNTs to carbon dioxide.

CNTs may be functionalized to enhance biodegradation by macrophages. This involves oxidation with mixed acids, commonly employed to increase CNT dispersibility or carboxylate CNTs (Yang and Zhang, 2019; Prajapati et al., 2022). Oxidized or carboxylated CNTs exhibit improved degradability by both enzymes and macrophages compared to pristine ones, likely due to the introduction of active groups (e.g., -OH and -COOH) or structural damage during the oxidation process, facilitating oxidative metabolism in cells. Hydroxylated SWCNTs show higher degradation rates than acid-oxidized SWCNTs and pristine SWCNTs, with the degree of incorporated defects influencing the degradation rate (Hou et al., 2016). Peroxidase-catalyzed degradation is more efficient with oxidized SWCNTs, aligning with positively charged arginine residues in the enzyme’s heme site. Covalent functionalization, such as linking azido coumarins and catechol derivatives to MWCNTs, has been explored as another approach (Sureshbabu et al., 2015). Studies demonstrated that designed substrates, including purine-modified MWCNTs, could effectively accelerate CNT degradation. A long-term comparative study on chemically functionalized MWCNTs (carboxylated, aminated, and both carboxylated and aminated) in primary microglial cells illustrated that surface functionalities play a crucial role in enzymatic biodegradability, suggesting strategies for the artificial design of biodegradable CNT materials (Bussy et al., 2013).

CNT cytotoxicity remains a topic of interest for many researchers as they seek to maximize the positive aspects of CNT while minimizing potential oxidative stress and cytotoxicity. To improve on current research in CNT toxicity, attention should be devoted to CNT functionalization. As seen earlier, one method to limit CNT toxicity is to limit CNT entry into the cells in the first place; this can be achieved by modifying CNT with polar groups. Alternatively, the noncovalent or covalent functionalization of CNT with biocompatible, or potentially bioactive, molecules can offset any negative cytotoxic effects of CNT. Finally, another approach that should be explored is multi-material 3D printing, which could encapsulate CNT-scaffolds within a more biocompatible exterior layer. Thus, the mechanical properties of the scaffold would be preserved while ensuring cell viability is not negatively affected. These proposed strategies would accelerate the implementation of CNT-based scaffolds *in vivo*.

## 4 CNT-based scaffolds in cancer therapeutics

Current CNT applications in targeted cancer therapy revolve around drug delivery. As easily functionalizable compounds, CNTs can be cross-linked with genes, proteins, or drugs to form complexes. These solutions are then injected into tumors, acting as drug delivery systems with enhanced membrane permeability due to the hydrophobic nature of CNT. However, this review will focus not on CNT drug delivery systems, but rather CNT-based scaffolds themselves. This section will explore how the unique physical properties of CNT lend themselves to unique applications such as phototherapy and electrothermal ablation that may be less invasive and more efficacious than current CNT-based drug delivery systems. For instance, a CNT-scaffold may take advantage of the inherent cytoxocity of CNT discussed earlier. While CNT cytotoxicity is undesirable in the context of tissue regeneration and proliferation, such effects may be beneficial in the area of cancer therapeutics. Combined with the unparalleled customization aspect of 3D-printing, scaffolds can be tailored to selectively target cancer cells for apoptosis. A recent study investigated the interaction between a collagen-carbon nanotube (Col-CNT) matrix and SKOV3 ovarian cancer cells (Li et al., 2021). Notably, the induced cells in the Col-CNT matrix exhibit reduced expression of CD44 (cell-adhesion glycoprotein) and increased expression of MMP2 (matrix metalloproteinase-2, a collagenase), making them less aggressive and more responsive to the chemotherapy drug gemcitabine. These findings suggest that the col-CNT matrix drives the transdifferentiation of epithelial cancer cells into a state of reduced aggression and potency, key to overcoming chemoresistance and tumor metastasis.

One study synthesized a gelatin network composed of CNT-COOH and sodium montmorillonite (Na-Mt) nanoparticles (Saber-Samandari and Toghraie, 2023). Through testing with lysozyme degradation studies, the scaffolds were tuned to possess similar degradation rates as native bone tissue regeneration. The porous scaffolds were irradiated with near infrared radiation and demonstrated high photothermal efficiency, with temperatures reaching greater than 43°C within 10 s. Unlike many other conductive nanofillers, semiconducting CNTs can absorb near-infrared (NIR) light, leading to localized heating. This property can be utilized for photothermal therapy, where the CNTs are targeted to specific tissues and then irradiated with NIR light to induce localized hyperthermia for cancer treatment or tissue ablation. The photothermal effect is produced through the photoexcitation phenomenon when photon adsorption excites a quantum system. CNT specifically possess a large NIR absorbance coefficient (300%) and a high thermal conductivity (3000 W/mK), both of which heighten its photothermal efficiency (Neelgund and Oki, 2018; Lee et al., 2022). Other studies show that hypothermia at such temperatures is effective for eliminating tumor cells locally (Yallapu et al., 2011; Sainitya et al., 2015). Results indicated that the presence of Na-MT nanoparticles alone were not effective in absorbing radiation, and that the present of carboxylated CNT was key as a photothermal agent.

**FIGURE 12:**
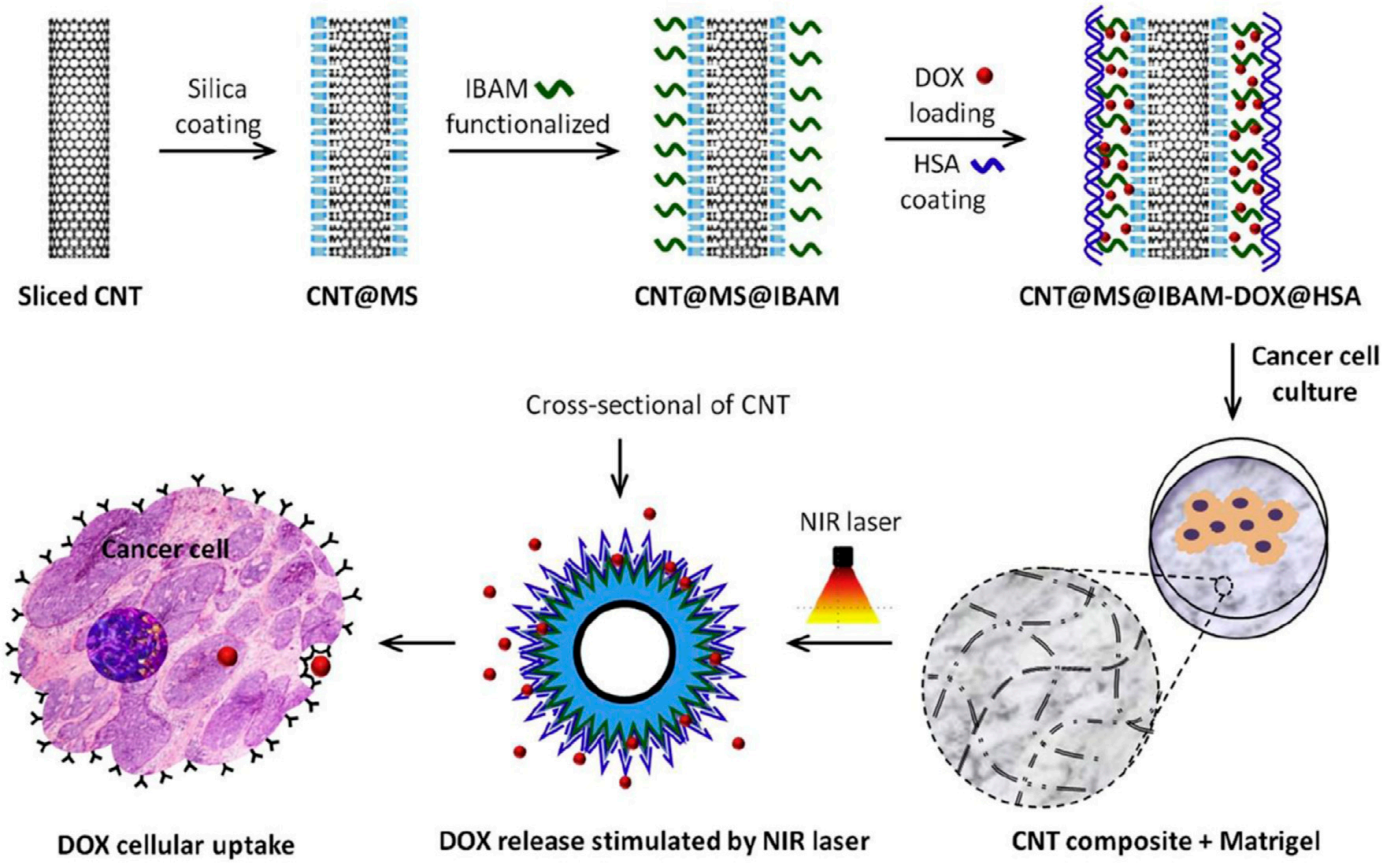
CNT scaffolds may act as a photothermally excitable trigger to release chemotherapeutic agents. DOX was loaded into a CNT-Matrigel composite. Stimulation of specific areas with NIR laser corresponded with DOX cellular uptake. Further, DOX uptake could be tailored to a burst or pulsatile fashion depending on the nature of NIR excitation. Reprinted with permission from Li et al. (2020a). Copyright 2020, Elsevier.

While phototherapy can be used alone, researchers have also combined the high photothermal conversion efficiency of CNT with its drug delivery properties. In another work, hybrid mesoporous silica-coated CNTs nanoplatforms were designed for combined phototherapy and controlled drug delivery (Li et al., 2020a). The release of the antitumor drug doxorubicin (DOX) from the nanocomposites was triggered only upon photothermal excitation, demonstrating a controlled drug release mechanism mediated by NIR laser excitation (Figure 12). Indeed, very little cytotoxicity was observed when no NIR was applied, indicating high specificity and selectivity. Moreover, while a release of DOX triggered moderate cellular death, it was only with the synergy of photothermia and DOX release that an efficient cytotoxic effect was achieved. The release profile of DOX could even be tailored to a sudden burst or pulsatile fashion, depending on the pattern of NIR irradiation. The customizability and efficacy of this study opens up possibilities for implantable antitumor scaffolds that can be easily triggered from outside stimuli to provide phototherapy at specific locations.

**FIGURE 13:**
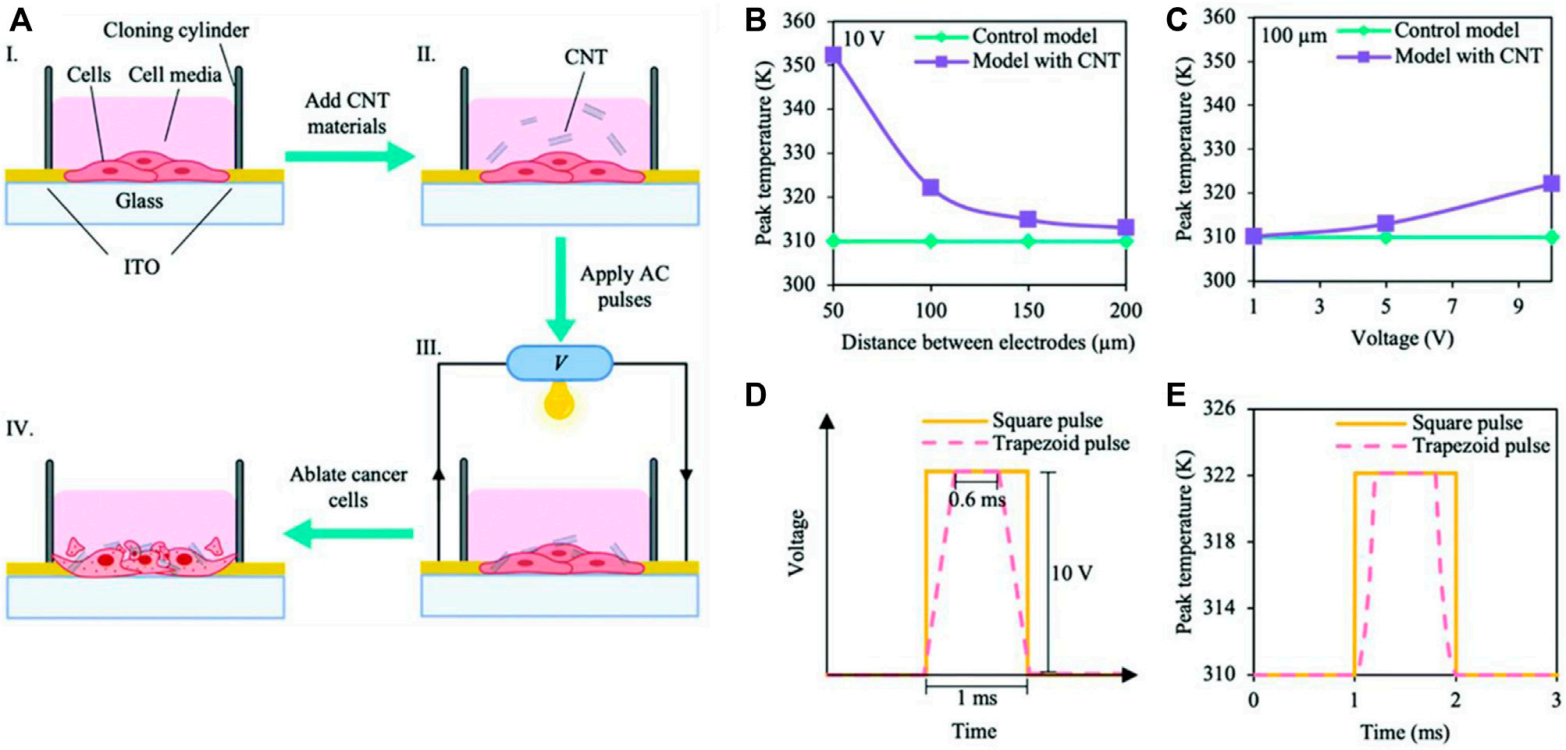
**(A)** An electrothermal therapy platform emits AC pulses to ablate cancer cells. **(B,C)** Simulated peak temperatures show increase in peak temperatures in models with CNT. Moreover, as distance between electrodes decreases, resistance decreases and the peak temperature increases. **(D,E)** Temperature profiles are dependent on the shape of electrical pulses. A square bias-voltage pulse (amplitude = 10 V, pulse width = 1 ms) and trapezoid bias-voltage pulse (amplitude of 10 V, pulse width = 0.6 ms) are tested. Reprinted with permission from Chan et al. (2022). Copyright 2022, Royal Society of Chemistry.

Electrothermal ablation through CNT-based platforms may even allow for targeted cancer cell destruction without the need for additional chemotherapeutic drugs. A study showed that, through enhanced Joule heating effects, alternating-current (AC) pulse CNT platforms lead to an increased peak temperature within cell layers when subjected to extended stimulation (Chan et al., 2022). AC pulses of amplitude 10V, pulse width 1 ms, and number of pulses = 10 were applied to the cells and cell death was observed (Figure 13). Electrothermal ablation is found to be enhanced in luminal breast cancer (MCF-7) cells while maintaining cell viability in healthy breast epithelial (MCF-10A) cells under the same conditions. The study found a ∼68% decrease in cell viability for the proposed system, significantly higher than the average ∼40% decrease across current photothermal nano-agents. Thus, CNT has emerged as a powerful standalone tool without the need for additional chemotherapeutic drugs. The proposed system is a promising start to efficient electrothermal cancer ablation treatments and demonstrates the clinical relevance of CNT platforms as an electrically conductive, bioactive tool.

CNT scaffolds are prime candidates for cancer treatment. Unlike previous studies on tissue regeneration, the cytotoxicity of CNT is less of a concern. Instead, attention is directed towards CNT’s strong Joule heating effects, high photothermal conversion efficiency, and drug delivery properties. All of these properties enable efficient, localized, and responsive cancer therapy solutions that surpass the solutions currently in practice. However, while CNT-based cancer therapy is well-studied, implementation of CNT-scaffolds *in vivo* is much less so. As of now, studies on CNT and its Joule heating effects rely on injections of CNT-based solutions into the tumor or injury site. This limits control over the extent of CNT diffusion and increases potential side effects on healthy cells. A promising solution lies in implantable CNT-based scaffolds, which can precisely control the area of cell death. Moreover, to assist with this technology, extrusion-based 3D-printing has emerged as a powerful tool to micro-engineer CNT-scaffolds in micrometer resolution (Shar et al., 2023). Future platforms could scan an external tumor such as a melanoma, design an appropriate scaffold with CAD software, and 3D-print a CNT-nanocomposite scaffold. The scaffold can be positioned snugly over the tumor site and photothermal therapy can then be induced accurately and effectively.

## 5 Conclusion and perspectives

Carbon nanotubes are versatile tools in bioengineering, providing an array of desirable properties to the scaffold at hand, such as increased electrical conductivity, mechanical properties, and cell adhesion. Across a broad range of body systems, from nervous tissue to cardiovascular and bone tissue, CNT scaffolds promote stem cell attachment, elongation, and differentiation. In conjunction with CNT scaffolds, strategies such as electrical stimulation and magnetic stimulation heighten the impact of electrical conductivity on the cellular microenvironment, further increasing cellular growth. CNT-based scaffolds offer a distinctive advantage in addressing challenges within neural tissue engineering. Vital processes for nerve regeneration, including axon regrowth, cell migration, and nerve cell attachment, gain from a conductive microenvironment. Moreover, EM stimulation has garnered attention as a potential strategy to amplify cellular response and regeneration in neural tissue engineering. This approach draws from the natural generation of electric fields during wound healing. By applying an electric field through a scaffold, the healing process is simulated, fostering nerve growth and sprouting. These effects, fundamental to neuronal migration and plasticity, offer insight into how electric fields facilitate nerve regeneration and underscore their significance in the field of neural tissue engineering.

In recent years, significant progress has been made in employing CNT scaffolds for cardiac and muscle tissue engineering. However, opportunities for enhancement remain, including the integration of machine learning to design micro-physiological systems. This approach would enable rapid analysis and fabrication of scaffold geometry and cellular responses, streamlining efforts to optimize survival rates. Additionally, exploring genome editing as a means to enhance immune resistance and robustness of implanted cardiomyocyte cells is crucial. In the realm of bone tissue engineering, it is important to extend research beyond CNT alone and explore innovative nanocomposites incorporating various materials. Future research could revolve around CNT-metal composites that combine the strengths of both elements. CNTs also offer the advantage of tunable mechanical properties through varying CNT loading percentages, as well as enhanced protein adhesion. By capitalizing on these benefits along with the biocompatibility of titanium, the potential arises for the creation of next-generation bone implants.

While CNT are attractive nanomaterial fillers for tissue engineering scaffolds, development is still needed to address several limitations. The processing technology for CNTs, given their insolubility in water and most organic solvents and tendency to agglomerate, remains a challenge. This makes it difficult to disperse them evenly in the polymeric matrix, affecting rheological behavior, especially with high CNT concentrations. New directions in improving homogeneity at concentrations above 5%–10%, in polar polymers, and without harmful solvents should be explored. While traditional organic solvents are still commonly used, there is a growing interest in environmentally friendly alternatives like 1,3-dioxolane and ionic liquids for safer mixing. Molecular dynamic simulations are emerging as a valuable tool to understand CNT dispersion on a molecular level, aiding in identifying suitable surfactant and solvent combinations for efficient dispersion. Moreover, the electrical conductivity inherent to CNTs presents an intriguing avenue for leveraging their potential in stimulating tissue growth and repair. The application of electrical cues can significantly influence cellular behavior and provide guidance in tissue regeneration processes. Customized CNT scaffolds with precisely controlled conductivity could be tailored to suit specific applications, effectively harnessing the electrical properties of CNTs for therapeutic purposes.

The focus of research community remains on biocompatibility, a core objective aimed at mitigating potential adverse reactions caused by CNTs within the body. Techniques involving alterations to the surface and functionalization are being actively explored to enhance the harmonious interaction of CNTs with the surrounding tissues. For example, scientists work on refining the biocompatibility of CNTs through various surface modification methods. These efforts encompass techniques such as applying polymer coatings, functionalizing with biocompatible molecules, or modifying the surface charge of CNTs. By tailoring the surface characteristics of CNTs, the intent is to diminish the potential for undesired reactions and elevate the overall safety of materials founded on CNTs.

Furthermore, research into bioactive coatings is ongoing with the purpose of enveloping CNT scaffolds with bioactive molecules. This tactic is designed to fortify cell adhesion, proliferation, and differentiation, in a manner that closely mirrors the natural environment of cells. This in turn fosters an enhanced fusion of engineered tissues with the surrounding biological matrix. For example, one avenue involves coating CNT scaffolds with growth factors or signaling molecules that exert pivotal roles in cell adhesion, proliferation, and differentiation. These bioactive coatings effectively function as guides, steering cells to behave in specific ways, much akin to their behavior in their native environment. To illustrate, CNT scaffolds could be coated with a bioactive protein that prompts stem cells to differentiate into bone-forming cells. This in turn facilitates a more efficient integration of the scaffold with the adjacent bone tissue. These nanoparticles possess the capacity to release therapeutic agents like antibiotics or growth factors over time, thereby supporting tissue regeneration and curtailing the risk of infections. The incorporation of such bioactive elements into the scaffold design is intended to cultivate an environment that stimulates the desired cellular responses and accelerates the healing process. These strategies not only refine the compatibility of materials with the human body but also actively guide cellular behavior, culminating in better tissue integration and functional outcomes.

Moreover, it is important to take into account the potential cytotoxicity of CNT. Strategies such as functionalization of CNT and decreasing percentage loadings can reduce cytotoxicity while maintaining positive effects on conductivity and mechanical strength. Modifying CNTs with polar groups can limit their entry into cells, reducing toxicity. Additionally, noncovalent or covalent functionalization with biocompatible or bioactive molecules can counteract cytotoxic effects. Exploring multi-material 3D printing presents another avenue by encapsulating CNT scaffolds in a biocompatible exterior layer, preserving scaffold mechanics and promoting cell viability. These strategies hold the potential to expedite the safe integration of CNT-based scaffolds *in vivo*. In some cases, cytotoxic effects can be capitalized on as routes to next-generation cancer therapeutics. Recent discoveries in CNT-based electrothermal ablation and phototherapy show promise in cutting-edge cancer cell treatments with muted effects on healthy cells. Current investigations into CNT’s Joule heating effects typically involve injecting CNT-based solutions into tumor or injury sites. However, this approach lacks precise control over CNT diffusion and may lead to unintended effects on healthy cells. A promising alternative involves the use of implantable CNT-based scaffolds, offering meticulous control over the region of cellular damage. Navigating regulatory considerations is of paramount importance when introducing innovative materials like CNTs into medical applications, ensuring the utmost safety of patients. Subsequent research endeavors are likely to involve collaborative efforts with regulatory agencies to establish comprehensive guidelines and safety standards governing the use of CNT-based tissue engineering products.

Additionally, extrusion-based 3D printing has emerged as a valuable tool for micro-engineering CNT scaffolds with high resolution. This technology envisions a future where external tumors, such as melanomas, can be scanned, suitable scaffold designs generated using CAD software, and CNT-nanocomposite scaffolds 3D-printed accordingly. These scaffolds could be precisely positioned over tumor sites, facilitating accurate and effective photothermal therapy induction. Ultimately, CNT scaffolds will continue to be important assets in the search for conductive and biocompatible tissue engineering models. In terms of scaling up and manufacturing, the viability of CNT scaffolds as a commercially viable solution hinges on the development of scalable manufacturing processes. These processes must be capable of consistently producing high-quality scaffolds suitable for clinical applications.

An essential challenge lies in designing CNT scaffolds that actively promote the formation of blood vessels (vascularization) within engineered tissues. Researchers are exploring innovative methods, such as incorporating growth factors or creating specialized structures within CNT scaffolds, to facilitate the development of a functional vascular network. The progression from laboratory settings to *in vivo* studies is a pivotal step in comprehending the long-term effects and interactions of CNT scaffolds within living organisms. This journey could ultimately lead to clinical trials, assessing the efficacy and safety of CNT-based tissue engineering strategies in human subjects. Furthermore, as the field of tissue engineering evolves, personalized approaches employing CNT scaffolds are likely to become more prevalent. Tailoring scaffold properties to align with individual patients’ needs has the potential to yield more successful tissue integration and regeneration.
